# Dynamics of dendritic cell maturation are identified through a novel filtering strategy applied to biological time-course microarray replicates

**DOI:** 10.1186/1471-2172-11-41

**Published:** 2010-08-03

**Authors:** Amy L Olex, Elizabeth M Hiltbold, Xiaoyan Leng, Jacquelyn S Fetrow

**Affiliations:** 1 Department of Computer Science, Wake Forest University, Winston-Salem, NC 27109, USA; 2 Department of Microbiology and Immunology, Wake Forest University School of Medicine, Winston-Salem, NC 27157, USA; 3 Department of Biostatistical Sciences, Division of Public Health Sciences, Wake Forest University School of Medicine, Winston-Salem, NC 27157, USA; 4 Department of Physics, Wake Forest University, Winston-Salem, NC 27109, USA

## Abstract

**Background:**

Dendritic cells (DC) play a central role in primary immune responses and become potent stimulators of the adaptive immune response after undergoing the critical process of maturation. Understanding the dynamics of DC maturation would provide key insights into this important process. Time course microarray experiments can provide unique insights into DC maturation dynamics. Replicate experiments are necessary to address the issues of experimental and biological variability. Statistical methods and averaging are often used to identify significant signals. Here a novel strategy for filtering of replicate time course microarray data, which identifies consistent signals between the replicates, is presented and applied to a DC time course microarray experiment.

**Results:**

The temporal dynamics of DC maturation were studied by stimulating DC with poly(I:C) and following gene expression at 5 time points from 1 to 24 hours. The novel filtering strategy uses standard statistical and fold change techniques, along with the consistency of replicate temporal profiles, to identify those differentially expressed genes that were consistent in two biological replicate experiments. To address the issue of cluster reproducibility a consensus clustering method, which identifies clusters of genes whose expression varies consistently between replicates, was also developed and applied. Analysis of the resulting clusters revealed many known and novel characteristics of DC maturation, such as the up-regulation of specific immune response pathways. Intriguingly, more genes were down-regulated than up-regulated. Results identify a more comprehensive program of down-regulation, including many genes involved in protein synthesis, metabolism, and housekeeping needed for maintenance of cellular integrity and metabolism.

**Conclusions:**

The new filtering strategy emphasizes the importance of consistent and reproducible results when analyzing microarray data and utilizes consistency between replicate experiments as a criterion in both feature selection and clustering, without averaging or otherwise combining replicate data. Observation of a significant down-regulation program during DC maturation indicates that DC are preparing for cell death and provides a path to better understand the process. This new filtering strategy can be adapted for use in analyzing other large-scale time course data sets with replicates.

## Background

Today's technological advances have provided biomedical researchers with an abundance of information, especially in the field of molecular biology. High throughput technologies, such as microarrays, are capable of generating large volumes of data in a short period of time. These technologies provide the unique opportunity to study the temporal dynamics of biological processes in a global fashion rather than one gene or small groups of genes at a time. However, studying temporal dynamics adds another dimension to data that is already large scale—that of time. Even without this additional dimension, the development of methods for the filtering, organization and analysis of these large data sets is an active area of research and presents a major hurdle for biologists [1,2].

Time course experiments are designed to observe the temporal dynamics of a particular biological process. A good example of such a process is the maturation of dendritic cells (DC), an important cohort of cells that serve as sentinels of the immune system. As reviewed in Banchereau *et al *and Guermonprez *et al *[3,4], these cells sense and respond to pathogens and inform the adaptive immune system on the nature of the foreign invader. Upon interaction with pathogens or their components, DC undergo a transformation process known as maturation. Through this process, their ability to stimulate the immune responses is enhanced; thus, these cells are critical initiators of the adaptive immune response.

The well characterized cellular processes associated with DC maturation include, but are not limited to: up-regulation of co-stimulatory molecules and inflammatory cytokines, down-regulation of endocytic/phagocytic activity facilitated by changes in rates of membrane turnover and changes in cytoskeleton, changes in cell morphology and migration due to up-regulation of chemokines, chemokine receptors and adhesion molecules, and increases in degradative capacity associated with down-regulation of protease inhibitors [5-12]. DC maturation is also a terminal differentiation process marked by shut down of the cell cycle followed by the eventual programmed death of the cell [13-16].

DC maturation is a highly complex, time-ordered process involving changes at many levels including gene expression, intracellular transport, cytoskeletal activity, and localization within the host. A dynamic process of interaction among gene transcripts, regulatory sequences, and trans-acting factors creates an underlying gene expression network that is extremely important for controlling many of the observed changes that occur during the process of DC maturation. A number of time course studies of the process of DC maturation induced by different stimuli have been published [17-21], providing significant insight into the dynamics of this process.

Biological data is, by nature, 'noisy'; that is, there are many points during the experimental and analysis processes where biological and technical variations are introduced [1,22]. Replicate experiments reduce the effect of these variations on the results, and help ensure that reported results are reproducible. Feature selection methods must be able to identify those genes that were reproduced in replicate microarray experiments. DC time course maturation studies [17-21] evaluate replicate data in different ways. Most often, the significance of expression of each gene in each replicate is evaluated individually, and then the expression signals are averaged before clustering. In some studies, only one replicate is used for clustering. In all cases, time points are treated individually, thus these studies do not fully utilize the time-dependent information inherent in these replicate time course experiments.

Some of the most popular feature selection processes for analysis of microarray data employ complex statistical methods such as Analysis of Variance (ANOVA) [23] and Significance Analysis of Microarrays (SAM) [24,25] to identify those genes that were significantly expressed. Others perform feature selection through simpler statistical methods involving t-statistics or other techniques [26] such as selecting ad hoc fold change thresholds. Most of the statistical methods use the variance within arrays and between replicate arrays to determine which genes are significantly expressed above the statistical noise (i.e. for time course experiments they consider each time point in isolation of the others). These techniques work well for all types of microarray experiments and have been shown to elucidate biologically significant gene transcripts [25,27,28]; however, the dynamic time-dependent information may not be fully utilized.

Because time course experiments provide an additional dimension for feature selection methods to consider, consistency in *scale *and *pattern *of response across a time course profile, not just at a single measurement, must be assessed. Here, we aim to develop a filtering strategy that explicitly identifies consistency in scale and pattern of time course replicate data, and we apply this process to a DC maturation microarray time course experiment.

Beyond the selection of significant gene expression profiles, time course data must be further organized. Clustering is an important exploratory tool that aids in the analysis and organization of biological data by dividing the data set into smaller, more manageable groups based on some definition of similarity. Analysis of differentially expressed gene clusters can reveal functionally related genes [29,30] or genes that may have the same regulatory mechanism [29,31]. This popular analysis technique is generally performed on a single data set using traditional clustering algorithms, such as hierarchical agglomerative clustering (HAC), k-means, or self-organizing maps [29]. Consensus clustering is another strategy that aims at identifying highly robust clusters in a data set. This is generally done using a bootstrap technique [32] with one clustering algorithm or by taking a consensus over many different clustering algorithms [33].

An issue that surfaces during any cluster analysis is that of reproducible results between replicate experiments. A comparison analysis done by Yeung *et al *concluded that incorporation of replicate expression data into clustering produces clusters with higher stability [34]. Various techniques have been implemented to incorporate replicate gene expression data into clustering. These methods generally use replicate data to assess the variability of individual measurements and incorporate this error information into the similarity metric or directly into the clustering algorithm [34-37]. These methods frequently combine replicate values (often by averaging) for the final clustering [34,36,37]; however, if clustering is performed on one replicate, or the other, or the average, independently, different results may be returned. This was observed in our own experiments where clustering each replicate experiment independently resulted in one replicate being dominated by up-regulation and the other by down-regulation. We expect some variation in biological replicate experiments; however, we also expect the bulk of the data to be reproducible, so that similar biological conclusions are reached in both experiments.

In this work we aim to identify key features in the temporal dynamics of gene expression during DC maturation following poly(I:C) stimulation through the implementation of a new filtering strategy. Many available methods are designed for filtering and clustering time course data [38-40]. Our filtering and clustering strategy emphasizes the importance of reproducibility between replicate experiments in both the feature selection and clustering. Our feature selection strategy utilizes time course profiles, rather than isolated time points, to identify genes that respond and behave consistently to stimuli over time in replicate experiments. We then apply a modified consensus clustering approach to obtain robust gene clusters from replicate data sets. Many of the previously known aspects of the DC maturation process were observed here, supporting the validity of our method. In addition, several novel patterns of gene expression related to signaling and transcriptional regulation are reported, highlighting the potential of the method.

## Results and Discussion

The Results and Discussion section is organized as follows. First we describe the results achieved from application of each step of the filtering and analysis process (methodological details of the process are presented in the Methods section). Next, we demonstrate why this method is important to obtaining reproducible data. We then report on how our microarray data compares to a similar time course microarray experiment, followed by an overview of our results with comparison to the literature. Finally, a detailed evaluation of the observed dynamic process of DC maturation is provided.

### A filtering strategy that identifies those genes and gene clusters that are highly consistent across two replicate experiments

#### Feature Selection

**Figure 1:**
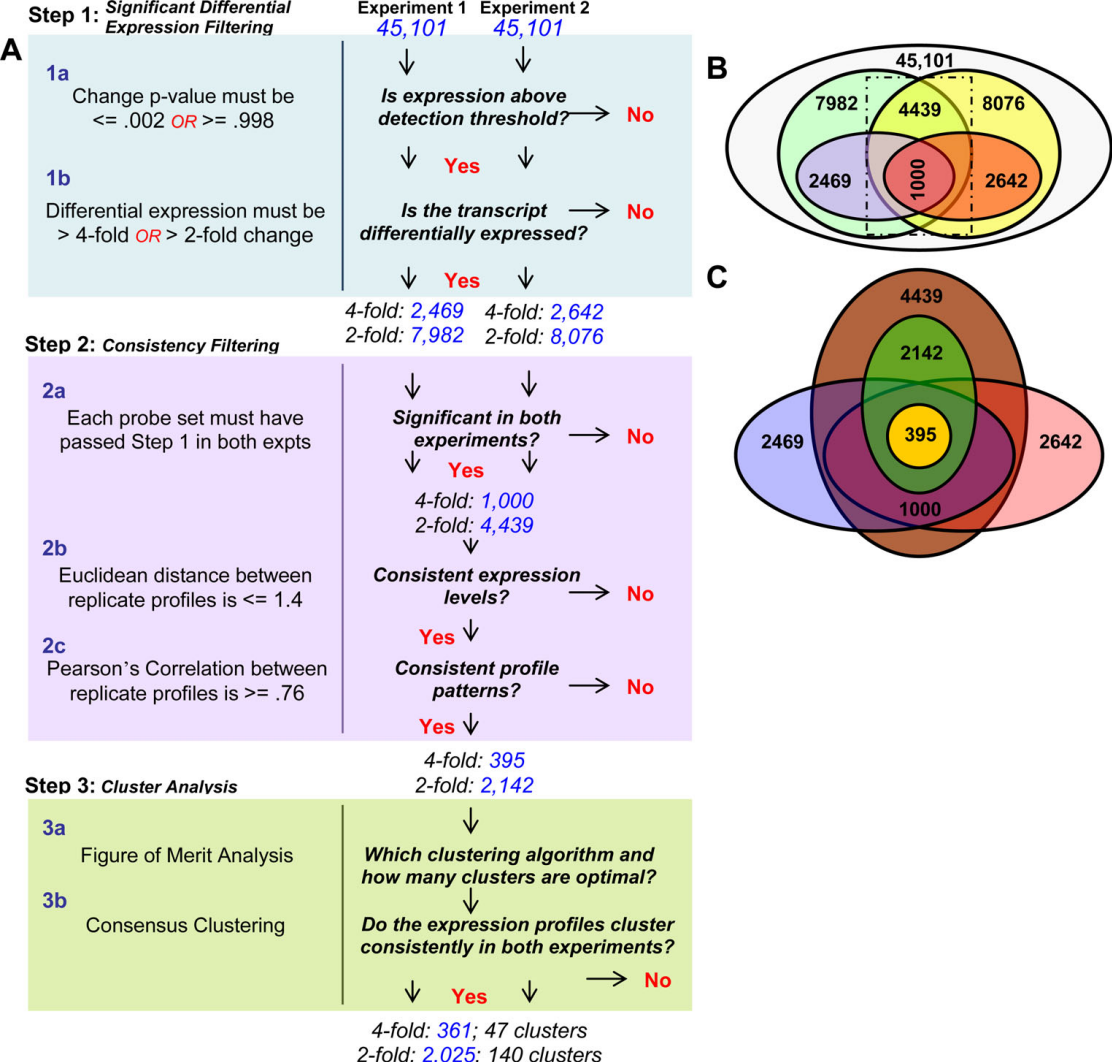
**Outline of feature selection and cluster analysis process**. A flow chart of the analysis process is shown in (A) and is composed of 3 steps. Step 1 is the *significant differential expression filtering *where each gene was required to have at least one time point where the signal's change p-value and the fold change met the indicated criteria. Step 2 is the *consistency filtering *of the results from Step 1 where genes that exhibit consistency of expression level and profile pattern between experiments are identified. Step 3 is the *cluster analysis *of all data from Step 2 and includes a FOM analysis followed by consensus clustering. (B) A representation of all datasets generated from Step 1 in (A) and their overlaps. The gray circle represents all 45,101 genes contained on the Affymetrix GeneChip; the green (7982) and blue (2469) circles on the left are the number of probe sets that met the 2-fold and 4-fold filtering criteria respectively for experiment 1; the yellow (8076) and red (2642) circles on the right represent the number of probe sets that met the 2-fold and 4-fold criteria respectively for experiment 2. (C) An expanded view of the dotted box in (B) showing the results of Step 2 in (A): the purple (1000) and brown (4439) circles represent the common gene lists for the 4-fold and 2-fold criteria between both experiments (step 2a). The inner yellow (395) and green (2142) circles represent the two final sets of genes that were determined to have the most significant and consistent differential expression for the 4-fold and 2-fold filtered data, respectively.

In the feature selection phase of the analysis, the data from both replicate microarray experiments (called experiments 1 and 2; see methods) were subjected to a two-step filtering process (Figure 1A, Steps 1 and 2). This was done to ensure that the reported results included only significant differentially expressed (Step 1) and consistent (Step 2) genes from each experiment.

Step 1 of the filtering process was performed on each experiment independently, and was designed to identify genes differentially expressed above a specific detection threshold for at least one time point (Figure 1A steps 1a and 1b; see methods for details). At the 2-fold level this resulted in an 82.3% and 82.1% decrease in the total number of genes under consideration for experiments 1 and 2, respectively (Figure 1B, green and yellow circles, respectively). Likewise, filtering at the 4-fold level decreased the number of genes by 94.5% and 94.1% for experiments 1 and 2, respectively (Figure 1B, blue and red circles, respectively). After completing this differential expression filtering step, four *gene lists *remained—one 2-fold and one 4-fold filtered gene list for each of the two replicate experiments.

Step 2 of the filtering process was designed to identify those detectable differentially expressed genes (those from Step 1) that also consistently responded to stimuli in both replicate experiments across all time points (see Methods). For an expression profile to be considered *consistent *between experiments, it must have: 1) been above the detection threshold and differentially expressed in both experiments (i.e. passed Step 1 in both experiments); 2) exhibited a consistent level (or scale) of response over time in both experiments; and 3) exhibited a similar expression pattern over time in both experiments. Therefore, Step 2 was implemented in three stages (Figure 1A, Step 2).

In step 2a the intersection of the gene lists from Step 1 at each fold change level was identified. There were 4,439 genes common to both experiments at the 2-fold level (Figure 1B, intersection of green and yellow circles; Figure 1C, brown circle), and 1,000 genes at the 4-fold level (Figure 1B and 1C, intersection of blue and red circles). Each identified gene list has two associated *data sets*, one from each experiment. Note that replicate data were not combined; this step merely identified genes that had a detectable significant differential expression at some point in both experiments.

Steps 2b and 2c (Figure 1A) used the calculation of Euclidean distance (ED) and Pearson's correlation coefficient (PCC) between replicate profiles to measure the consistency of expression level and profile pattern across analyzed time points, respectively (see Methods). While use of only ED or PCC is appropriate for some questions, utilization of both criteria ensures identification of the temporal profiles consistent in both scale and pattern across both data sets (see Additional file 1 for an example). Steps 2b and 2c resulted in a 51.7% and 60.5% reduction in genes for the 2-fold and 4-fold filtered gene lists, respectively (Figure 1C, green and yellow circles, respectively).

Overall, the two steps in the feature selection process reduced the total 45,101 genes on the chip to 2,142 genes (95.3%) for the 2-fold filtered and 395 genes (99.1%) for the 4-fold filtered data. These genes are considered to have a detectable, significant differential expression and their temporal profiles were consistent in both shape and scale.

A common approach to replicate data has been to average the data points and calculate a standard deviation or similar statistic for each data point [18,20,34,36,37,41]. Many studies average replicate experiment data for visualization [21], or as part of the process in identifying differentially expressed genes [18,20,41]. Averaging alters the data and could result in expression profiles that may not accurately represent the original. Several examples provided in Additional file 2 demonstrate how the average expression profile is different in both expression level and pattern from either of the replicate expression profiles used in the calculation. The 2-step feature selection method used in this study does not require averaging of data for either visualization or analysis, and it ensures that each expression profile exhibits a consistent pattern and scale across the time course. Thus, the list of significantly regulated and consistent genes obtained from Step 2 are similar enough that related biological conclusions should be reached no matter which experiment is used for visualization.

#### Consensus Clustering

Feature selection was followed by cluster analysis (Figure 1A, Step 3), which was performed in two stages, a and b. Step 3a is a preliminary analysis of all data sets to determine the optimal clustering schema (clustering algorithm and number of clusters) and step 3b is a modified version of consensus clustering.

**Figure 2:**
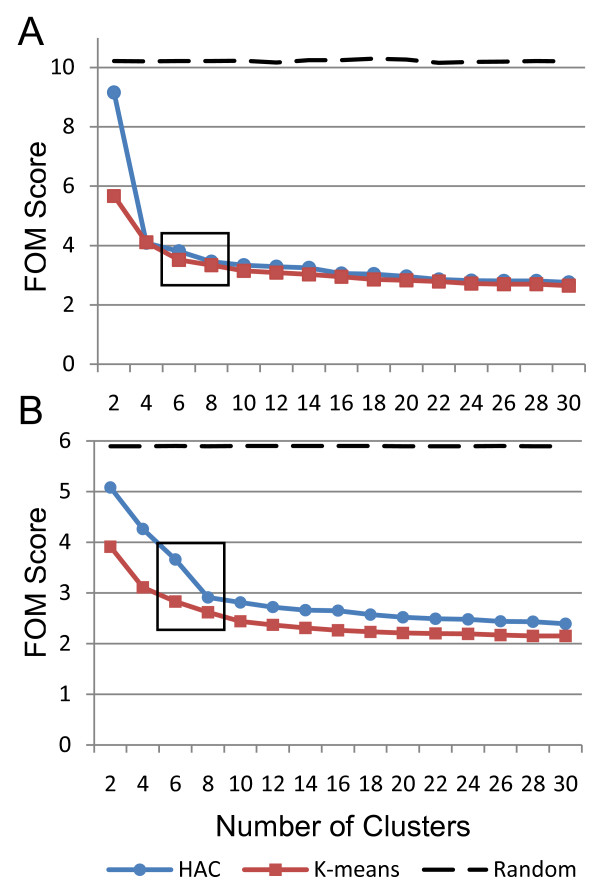
**Figure of merit analysis on both significant datasets indicates optimal clustering parameters**. The FOM scores over a range of cluster numbers for the 4-fold filtered data (A, 395 genes) and the 2-fold filtered data (B, 2142 genes) have been graphed. Both (A) and (B) were generated using the data from experiment 1 (experiment 2 exhibited similar results; not shown). This analysis indicates that k-means is the optimal clustering algorithm for both datasets since it has the lowest average FOM score (though this was not significant for the 4-fold data). The box on each graph indicates the optimal range for the ideal number of clusters is between 6 and 8; 8 was chosen as optimal for both data sets.

Step 3a uses the figure of merit (FOM), an internal validation measure that aids in quantitatively determining the optimal clustering schema [42,43]. The chosen schema optimizes cluster homogeneity with respect to the distance measure used. This is necessary because different underlying structures in the data are revealed depending on the clustering schema chosen. Various internal validation methods have been designed to find the best approximation of the natural cluster structure for biological data without imposing *a priori *biological knowledge on the data; however, many studies neglect to perform this step. Assessing the natural cluster structure of biological data is now being recognized as an important part of microarray data analysis [44]. In a recent study by Giancarlo *et al *[45], several of the more popular internal validation measures, such as FOM, Clest [46], Gap Statistic [47], Model Explorer [48] and others, were compared. Results showed that while FOM was not the overall best on computational time it did have good accuracy in predicting the correct number of clusters from a "gold standard" model. Since the filtered data sets in this study were small and computation time was not an issue, we used FOM to determine the optimal clustering schema [43]. For all the 2-fold and 4-fold filtered data sets, FOM analysis indicated that using 8 clusters and k-means was the optimal schema for this data (Figure 2).

In the last step, 3b, consensus clustering was done to identify those groups of genes that consistently clustered together in both replicate experiments to form robust clusters. Because k-means is a non-deterministic clustering method, each replicate data set was clustered several times to generate multiple clustering solutions associated with each experiment; then a consensus was taken over all solutions to identify the robust clusters (see Methods). There are two important aspects of this consensus clustering approach: 1) standard clustering repetitions (for stochastic methods like k-means) on the same data set are used to identify groups of genes that form clusters within each experiment independently; and 2) the method identifies those sub-groups of genes that cluster consistently in replicate data sets. The first is important because it associates expression profiles that have highly similar patterns or expression levels without the bias of historical functional knowledge imposed on the clusters. The second identifies clusters of genes that respond to stimuli as a group even though the response may be different in each replicate experiment (Additional file 3 provides an example). Such clusters may be controlled by a common regulatory mechanism. Clustering methods that incorporate the error or variation between replicate data [34,36,37] may not identify these groups of genes because they ultimately combine replicate data into one solution. Consensus clustering across experimental conditions other than a time course could be used to identify groups of genes that consistently change together in response to different stimuli or in different tissues (such as diseased and normal), which might suggest underlying biological mechanisms. These proposed variations in consensus clustering will require further investigation to determine their full potential.

The application of consensus clustering on the filtered data sets resulted in a total of 140 (2-fold) and 47 (4-fold) consensus clusters with 2 or more genes per cluster (see Additional file 4: Tables S1 and S2 for a full list of consensus clusters). There were 117 genes from the 2-fold data set and 34 genes from the 4-fold data set that formed singleton clusters and were not included in subsequent analysis.

Overall, this filtering strategy resulted in more than a 95% reduction in genes under consideration and revealed a number of clusters, which were then used to explore the dynamics of DC maturation.

### A partial analysis would result in different and contradictory conclusions about the process of DC maturation

Our long term objective is to understand the molecular mechanisms underlying the temporal gene expression program of DC maturation. We argue that it is crucial to identify the most reproducible features across replicate experiments and that all steps shown in Figure 1A are important to achieving this goal. Stopping at any point in the analysis would yield different impressions of the DC maturation process.

**Figure 3:**
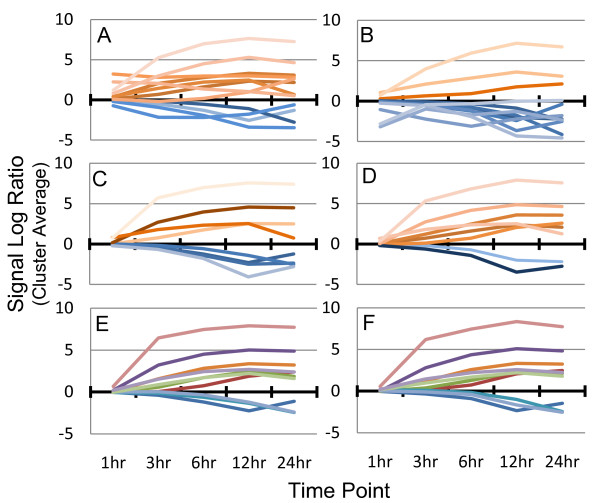
**Progression through the analysis process increases consistency of average cluster profiles**. Step 1 of the analysis process was performed on each experiment individually and the average profile for each cluster was plotted. The resulting data sets were analyzed with the FOM and clustered using k-means into 12 clusters. Experiment 1 (A) portrays the DC maturation process as mostly up-regulated (orange profiles), whereas experiment 2 (B) portrays it as mostly down-regulated (blue profiles). Next, Steps 1 and 2 were performed on experiments 1 (C) and 2 (D). The resulting data sets from each experiment were analyzed with the FOM and clustered using k-means into 8 clusters. These clusters are now more consistent across experiments, however, experiment 2 (D) is now showing DC maturation to be more up-regulated than experiment 1 (C). Finally, Step 3, consensus clustering, was performed on experiments 1 (E) and 2 (F) using the parameters defined in Methods. These clusters are now consistent across experiments. The 1 hour time point was not used for filtering or clustering, and is shown for reporting purposes only.

For example, if the data were filtered using only Step 1 followed by k-means clustering, then the resulting clusters for each experiment would portray the DC maturation process differently: as primarily up-regulated (experiment 1, Figure 3a) or primarily down-regulated (experiment 2, Figure 3b). Step 2 of the filtering process identifies and removes any gene that does not consistently respond to stimuli in both experiments. Thus, clustering these data provides a more similar view of the process of DC maturation (Figures 3c and 3d). However, even these clusters show differences between experiments: more up-regulated clusters in experiment 2 than in experiment 1. Performing consensus clustering (Step 3) on the filtered data sets (Figures 3e and 3f) refines our view even further by identifying genes that also consistently cluster together in both replicate experiments. In this comparison, it can be seen that the average cluster profiles are almost identical except for minor variations in expression at a few time points.

The graphs shown in Figure 3 illustrate the well-known concept that performing replicate experiments is important: if only one experiment was done then, depending on the data collected, different impressions of the DC maturation process would be obtained. These graphs also demonstrate that the full analysis to identify genes that are consistent in expression level, expression pattern, and clustering partners identifies temporal profiles that are consistent between replicates.

### Flow cytometry indicates a robust response to treatment with poly(I:C)

Before analysis, we demonstrated that poly(I:C) treatment does indeed cause a typical maturation response in DC. Upon poly(I:C) treatment of bone marrow-derived DC (BMDC), we typically observe a roughly 10-fold increase in the mean fluorescence intensity of CD86 expression over untreated DC along with strong increases in CD40, CD80 and MHC class II. We measured the cell surface expression of CD86 by flow cytometry at 24 hours post treatment. As depicted in Additional file 5, we observed a strong up-regulation of CD86 expression on virtually all of the DC 24 hours post treatment in experiment 1. This response was also observed in experiment 2 (data not shown). Therefore, these DC showed a robust maturation profile based on their cell surface phenotype.

### Dynamics of the DC maturation process induced by poly(I:C) are consistent with previously published data

**Figure 4:**
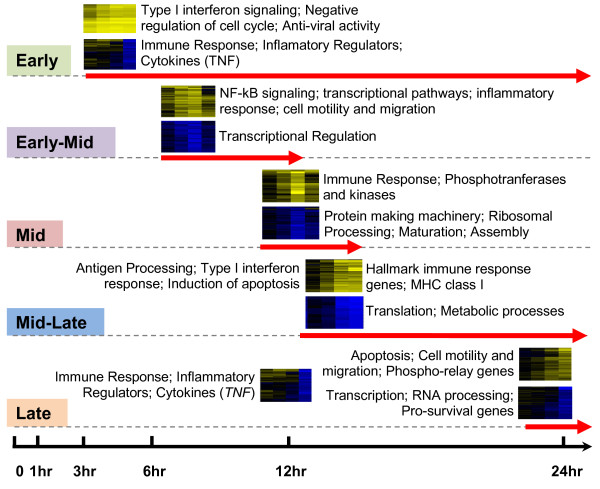
**Temporal ordering of clusters reveals progression of the DC maturation process induced by poly(I:C)**. The top 42 consensus clusters for the 2-fold filtered data set were manually placed into groups based on the visual expression profile exhibited by the heat map representation of each cluster. Five phases were identified for both the up- and down-regulated clusters: early, early-mid, mid, mid-late and late. These groups of clusters with similar expression patterns were ordered temporally to create a timeline. Each phase has one or more representative cluster heat maps, which indicate the patterns associated with that phase, and a red arrow indicating how long that phase lasted. A summary of the annotations included in each phase is provided next to each representative heat map (obtained from DAVID). Heat maps are formatted so that rows represent genes and columns are time points (3, 6, 12 and 24 hours); yellow indicates up-regulation, blue indicates down-regulation, and black indicates no change in expression relative to time zero. Phase assignments for each consensus cluster are included in Additional file 4: Table S2.

Five general phases of response were identified in the set of consensus clusters for the 2-fold filtered data: early, early-mid, mid, mid-late, and late. Grouping the consensus clusters based on these general phases, and ordering those groups temporally reveals the progression of the DC maturation process, involving both gene up-regulation and down-regulation, upon stimulation with poly(I:C). Figure 4 contains a summary of the five temporal phases with the associated functions and processes that were affected at each phase. To ensure our findings are consistent with previously published data, we compare expression profiles within each phase to a similar time course microarray experiment in the literature. We then discuss the similarities of our identified phases of response with previously defined phases of DC maturation.

**Figure 5:**
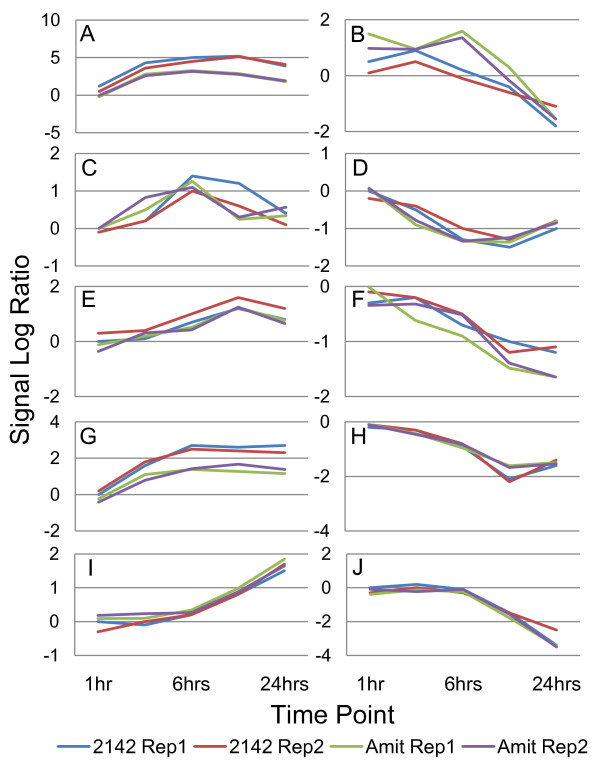
**Gene expression profiles are consistent with previously published microarray data**. Comparison to a similar experiment performed by Amit *et al *was done to ensure gene expression profiles were consistent with previously published data. Corresponding time points from the Amit *et al *data set were used to calculate Pearson's correlation coefficient between selected expression profiles from the 2-fold filtered data set. All graphs except one have a positive average correlation coefficient greater than 0.89; *Tle3 *(C) has an average correlation of 0.66. Figures are as follows: (A) *Ifih1 *(1426276_at) cluster 27; (B) *Fst *(1421365_at) cluster 11; (C) *Tle3 *(1419654_at) cluster 3; (D) *Gpr180 *(1417245_at) cluster 14; (E) *Clcn7 *(1450408_at) cluster 20; (F) *Siglecf *(1424975_at) cluster 2; (G) *Trim34 *(1426092_at) cluster 5; (H) *C1qbp *(1448274_at) cluster 17; (I) *Grina *(1436297_a_at) cluster 1; (J) *Fgf13 *(1418497_at) cluster 25.

Comparison of our microarray data to that of Amit *et al *[49] indicates a strong positive correlation of gene expression profiles on both a global and local scale. Amit *et al *performed a similar time course microarray experiment with 2 replicates using BMDC stimulated with poly(I:C) for 0.5, 1, 2, 4, 6, 8, 12, 16 and 24 hours. Scatter plots of data from Amit *et al *versus the corresponding data in this study show that on a global scale our data are positively correlated at each time point after 1 hour (Additional file 6). The 2,142 genes in the 2-fold filtered data set (which represents our most consistent data) were compared. Of the 2,142 genes, 1,641 were identified in the data provided by Amit *et al*. The array correlation between the genes in each replicate was above 0.7 in all cases, except the first time point (Additional file 6: Table S4). Examples of well correlated genes (PCC >= 0.89) between the two experiments from both up- and down-regulated clusters in each identified phase of response (Figure 5) show that the most highly correlated and consistent gene profiles in our data set are also highly correlated with the corresponding profiles reported by Amit *et al*. Thus, the data presented in this study are similar to published data on both a global and individual gene level.

The phases of DC maturation identified in this study (Figure 4) show similar characteristics to the common DC maturation response defined by Huang *et al *[5] (Additional file 7 shows a detailed comparison of gene annotations at each phase). Congruent with the literature, our experiments show that shortly after stimulation with poly(I:C), DC initiate significant up-regulation of anti-viral genes, type I interferon signaling, immune response genes such as the cytokine *Tnf-alpha*, and cytoskeletal genes. This early response is similar to the common maturation response of human monocyte derived DC stimulated by *C. albicans*, Influenza A virus or *E. coli *reported by Huang *et al *[5], which indicates that stimulation with poly(I:C) elicits many of the reported core maturation responses in DC. As 6 and 12 hours pass (middle phase), many signaling molecules are up-regulated along with transcriptional pathways and more inflammatory and immune response genes. After 12 hours (late in the process) the DC up-regulate antigen processing molecules, apoptosis genes, co-stimulatory and more signaling molecules, transcriptional regulators, and motility and migration genes. This again follows a similar pattern to those mid and late phases of the common DC maturation response reported by Huang *et al *where, after stimulation, the human DC increased expression of signaling genes, transcription factors, antigen processing and presentation genes, and migration-related genes.

Overall, the up-regulated expression program induced by poly(I:C) (Figure 4) exhibits many features to that of the common up-regulated program reported by Huang *et al*; however, the down-regulated program summarized in Figure 4 and Additional file 7 is more extensive than that previously reported. Between 6 and 24 hours genes associated with a large number of metabolic and catabolic processes are down-regulated, along with various cellular processes, cellular organization, binding, catalytic activity, and a large number of cellular component related genes. Thus, this more comprehensive down-regulated program may provide information on possible interplay between the up- and down-regulation that induces DC maturation. Both up- and down-regulated programs induced by poly(I:C) are now discussed in more detail.

### Differentially expressed genes are primarily observed late during the cell maturation process and are dominated by down-regulation

**Figure 6:**
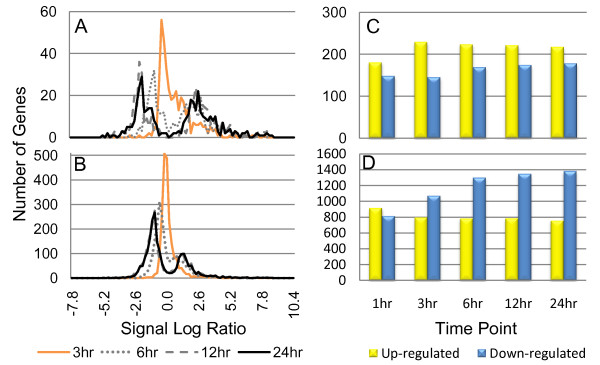
**The majority of transcripts are significantly expressed after 12 hours and are dominated by down-regulation**. The gene expression distributions at 3 (orange), 6 (gray dots), 12 (gray dashes) and 24 (black) hours for the 395 2-fold (A) and 2142 4-fold (B) filtered data sets for experiment 1 are portrayed as histograms (experiment 2 was similar; data not shown). The number of up- (SLR > 0) and down-regulated (SLR < 0) transcripts for the 2-fold (C) and 4-fold (D) filtered data sets were also counted at each time point to determine where up-regulation and down-regulation dominated the maturation process (experiment 2 was similar; data not shown). The number of transcripts with an SLR exactly equal to zero was not included in the bar graphs.

A global view of gene expression during the maturation process was obtained by plotting the distribution of gene expression at each time point (Figure 6). The 4-fold filtered (Figure 6a) and the 2-fold filtered (Figure 6b) data sets both exhibited a change in distribution from being unimodal at 3 hours to bimodal at subsequent time points. This result reflects a characteristic of the DC maturation process: the majority of those differentially expressed genes were not highly expressed or repressed until 12 to 24 hours following stimulation. Thus, many transcriptional regulatory processes and functions are not detectably affected by DC maturation induced by poly(I:C) until late in the process.

The histograms in Figure 6b also reveal another interesting observation: the bimodal distribution at 12 and 24 hours is skewed toward the negative side of zero, where no obvious skew is observed in Figure 6a. This indicates two things. First, more processes in DC maturation are down-regulated rather than up-regulated overall. Second, a large number of down-regulated genes at the later time points were significantly repressed at a level between 2- and 4-fold. A count of how many transcripts that were up- versus down-regulated at any given point reveals that if only transcripts induced or repressed by more than 4-fold are considered (Figure 6c), the maturation process is dominated by up-regulation. However, the down-regulated program becomes the dominant feature if we include transcripts induced or repressed by 2-fold or more (Figure 6d).

This result revealed two interesting characteristics about the genes involved in DC maturation: 1) there were relatively few transcripts that were highly expressed (395 with greater than 4-fold change was about 0.9% of the total array), however, the majority (58% at 3 hours) of those that were, were up-regulated (Figure 6c); and 2) when transcripts not so strongly expressed were observed (greater than 2-fold change), the maturation process became dominated by down-regulation in the later stages with 64% of the genes down-regulated by 24 hours (Figure 6d). These results suggest that many important characteristics of DC maturation might be found in the large numbers of genes that exhibit late down-regulation with expression changes between 2- and 4-fold. In the next several sections, we explore more explicitly which genes are up-regulated or down-regulated during DC maturation in the 2-fold filtered data set.

### The dendritic cell maturation "program": sustained temporal patterns of up-regulated gene expression

The gene expression patterns we observed over the 24 hour time course included many of the expected profiles such as up-regulation in immune response genes. There were three clusters of genes that were up-regulated quite strongly and remained so for the duration of maturation (Figure 4, early phase). These included clusters 22, 27, and 29, all of which exhibited strong induction (SLRs up to 2.5, 6, and 8 respectively). Not surprisingly, considering poly(I:C) is a strong inducer of the type I interferon response, many genes found in these clusters are involved in the type I interferon signaling and response pathways and/or exhibit anti-viral activity [50,51].

Cluster 29 (12 genes, 15 probe sets) is the most strongly up-regulated of the three clusters with genes up to 256-fold over-expressed. Analysis with the functional annotation clustering tool provided by DAVID [52] revealed one highly significant functional group in this cluster (p-value = 3.73 × 10-6). This functional group included 4 genes (6 probe sets) with a Gene Ontology (GO) (biological process) annotation of response to virus/other organism: *Irf7*, *Rsad2*, *Tgtp *and hypothetical protein LOC677168 (Affymetrix probe ID 1431591_s_at). Several of the genes in this cluster are important for the type I interferon response such as *Irf7*, which is an important transcription factor for the type I interferon signaling pathway [53], as well as *Iigp1*, *Isg20*, and *Rsad2 *which are target genes of the type I interferon response [54,55].

Cluster 27 contains 17 genes (18 probe sets) which are also strongly up-regulated (SLRs up to 6). Functional annotation clustering with DAVID did not identify any significant functional groups, however, several important and interesting genes were found in this cluster. These included the interferon activated genes, *Ifi47*, *Ifi203*, and *Ifih1 *as well as the anti-viral gene *Oas3 *[56,57]. The chemokine *Ccl5*, which is important for T cell recruitment [58-60], was also present. Interestingly, this cluster includes three members of the Schlafen family involved in negative regulation of the cell cycle, *Slfn1*, *Slfn3-4*, and *Slfn5 *[61-63]. Thus, even at early stages, the cell is beginning to shut down division and proliferation in favor of differentiation.

Cluster 22 includes 22 genes (23 probe sets) that are up-regulated (SLRs up to 2.5) at 6, 12, and 24 hours. Functional annotation clustering found no significant functional groups in this cluster, however, there are several genes involved in the immune response: *Tap2 *and *Psmb10*, which are important for peptide translocation and antigen processing, *Irak2 *and *Dok1 *which are involved in innate immune signaling, and *Cd47*, which is known to play an important role in maturation of specific DC subtypes [64-66]. We also found several other genes involved in intracellular transport such as syntaxin binding protein 1 (*Stxbp1*), and cytoskeletal reorganization genes including sarcoglycan beta (*Sgcb*) and microtubule-associated protein homolog (*Tpx2*).

Sustained up-regulation indicates that the processes represented by clusters 22, 27 and 29 are important throughout the duration of the maturation process. Thus, we hypothesize that this pattern of early and sustained up-regulation in the intracellular transport and cytoskeletal reorganization genes is important for the transport of MHC and co-stimulatory molecules to the cell surface for recognition by T cells and/or for the migration process to lymphoid organs.

### The dendritic cell maturation "program": genes that exhibit both up and down regulation

While most consensus clusters exhibited only up- or down-regulation, there were several genes identified by our analysis as significantly and consistently both up- and down-regulated at some point in the process. All of these genes clustered together in Cluster 11 (32 genes, 35 probe sets) and exhibit a pattern of early up-regulation followed by late down-regulation (Figure 4, early and late phase). While no significant functional groups were identified by DAVID, this cluster did contain the immune response cytokine, *Tnf*, as well as *Cxcl1*, a regulator of the inflammatory process. Other functions associated with this cluster include signal transduction, membrane transport, and one protein involved in response to reactive oxygen species (*Sod2*). Some of the protein products of these genes are often associated with the early events of DC maturation. This pattern of early up-regulation followed by significant and abrupt down-regulation at 24 hours suggests that these genes have potent effects, are tightly regulated, and may have deleterious effects if expressed in large quantities or over long periods of time.

### The dendritic cell maturation "program": temporal patterns of up-regulated gene expression

Aside from the sustained up-regulation of clusters 22, 27 and 29, several other patterns of up-regulation were revealed by the consensus clusters. For example, clusters 3 and 15 exhibit a pattern of peak up-regulation at 6 and 12 hours. This pattern is largely terminated by 24 hours (Figure 4, early-mid phase). Cluster 3 contains a large number of genes, 106 (125 probe sets), where significant (p-values < 0.05) functional group annotations include serine/threonine-protein kinases, phosphorylation, cell motility and migration, and response to wounding/immune response. Many of the genes included in cluster 3 are involved in the well characterized NF-kB signaling and transcriptional pathways such as *Tank*, *T2bp*, *Map2k1*, and NF-kB subunits epsilon and zeta (*Nfkbie *and *Nfkbiz *respectively). The RNA sensing pattern recognition receptor, toll-like receptor 7 (*Tlr7*), is also part of this cluster along with a number of inflammatory response genes such as interleukin 1 alpha (*Il1a*). There are many other signaling molecules, kinases and phosphatases, found in this cluster, including *Akt3*, *Ccnd1*, *Map2k1*, *Minpp1*, *Phlpp *and *Ppp2r5a*. These results suggest that much of the intracellular signaling associated with the DC maturation program begins within this "early to mid phase". Additionally, the program of cell motility and migration appears to be initiated in this phase as well. The 31 genes (34 probe sets) found in cluster 15 had a similar pattern of up-regulation in the middle phase of the program with a slightly higher magnitude of induction than cluster 3 (cluster 3 SLRs up to 2.5; cluster 15 SLRs up to 3.2). Significant (p-values < 0.05) functional group annotations for cluster 15 were different from the immune response oriented annotations of cluster 3 and included zinc finger B-Box and RING-type associated genes involved in transcriptional regulation. The genes identified in clusters 3 and 15 indicate that cell signaling and transcriptional regulation are important in the early-mid phase of DC maturation.

We observed one cluster of up-regulated genes, cluster 20 (24 genes, 27 probe sets), that exhibited peak gene expression only at the 12 hour time point (Figure 4, mid phase). This cluster contains the interleukin 13 receptor (*Il13ra1*), which is associated with the immune response, as well as *Atf3*, which is a transcription factor known to dampen innate immune signaling [67]. Among the functionally clustered genes identified by DAVID was a group of phosphotransferase/kinases (p-value = 0.04), none of which had previously been associated with the process of DC maturation: *Pftk1*, *Hk2*, *Rnasel*, *Brd2*, *Zcchc6*, and *Hif1a*. These proteins have generally been associated with metabolic pathways and intracellular transport. The significance of this particular group of genes to DC maturation remains to be determined, but provides an interesting cohort of novel genes for study.

A set of five clusters demonstrated a pattern of strong up-regulation later in the program at 12 and 24 hours including clusters 5, 6, 13, 32, and 34 (Figure 4, mid-late phase). Significant DAVID functional groups indicate that these clusters are enriched in immune response genes, and further investigation revealed that these clusters contained most of the "hallmark" genes known to be associated with DC maturation. Cluster 5 (64 genes, 73 probe sets) contained several highly significant DAVID functional groups with annotations that include MHC class I molecules (p-value = 2.95 × 10^-7^), SPRY and SPRY-like domains (p-value = 8.53 × 10^-5^), SAND family proteins (p-value = 4.37 × 10^-4^) and apoptosis/programmed cell death (p-value = 0.03). Genes that were part of the DAVID functional groupings were the co-stimulatory molecule *Cd86 *and two MHC class I molecules (*H2-T22 *and *H2-Q1*). This cluster also contained antigen processing molecules involved in the MHC class I processing pathway, such as *Tap1*, which were not part of a significant DAVID functional group. We also observed a strong type I interferon response, including *Ifi35*, *Stat1 *and *Oas1a*, and anti-viral gene induction. Other molecules of note in this cluster are those involved in the induction of apoptosis including caspase 4 (*Casp4*), caspase recruitment domain 4 (*Card4*), *CASP8 *and FADD-like apoptosis regulator (*Cflar*), Fas death domain-associated protein (*Daxx*) and clusterin (*Clu*).

Cluster 6 (51 genes, 54 probe sets), also one of the mid-late phase up-regulated clusters, contains a highly significant DAVID functional group (p-value = 1.02 × 10^-15^) that includes a probe set associated with several interferon alphas (1422403_at), as well as response to virus associated genes *Akt3 *and *H2-D1*. A second significant (p-value = 4.2 × 10^-3^) functional group is also found in Cluster 6 which includes the antigen processing TAP binding protein (*Tapbp*) and TAP binding protein-like (*Tapbpl*) genes. Likewise, the 30 genes (35 probe sets) found in cluster 13 include genes involved in the activation of T cells such as interleukin 18 (*Il18)*. Clusters 32 and 34 are composed of 13 genes (13 probe sets) and 10 genes (12 probe sets), respectively, that include a broad range of functional annotations such as immune response and antiviral activity as well as signaling, transcriptional regulation, and intracellular transport. Thus, most of the genes up-regulated in these mid-late phase clusters fall into expected categories based on what is already known about DC maturation.

Looking globally at these results, it is interesting to note that *Tapbp *(cluster 6) and *Tap1 *(cluster 5) are not up-regulated until late in the DC maturation process while *Tap2 *(cluster 22) had an early and sustained up-regulation. These three TAP gene products are crucial to the MHC class I antigen presentation pathway where TAP1 and TAP2 form a dimer that transfers peptides from the cytoplasm into the endoplasmic reticulum for loading onto the MHC class I molecules [68]. *Tapasin *(*Tapbp*) mediates the loading of peptides onto MHC molecules [69] where it is then processed and transported to the cell surface for activation of T cells. The up-regulation of *Tap1 *and *Tapbp *coincides with the timing of DC migration to the lymph nodes where T cell activation takes place, suggesting that the three proteins working together is important at that mid-late phase of maturation. The delayed up-regulation of *Tap1 *and *Tapbp *suggest that these may be the rate limiting molecules that facilitate higher levels of antigen presentation late in the maturation process. These TAP genes demonstrate how our filtering strategy is able to highlight interesting temporal dynamics within a small group of interacting proteins.

Cluster 1 is the only cluster whose genes are up-regulated at the latest time point (24 hours). It is a very large group of 225 genes (270 probe sets) whose expression is strongest primarily at 24 hours (Figure 4, late phase). In this cluster, DAVID analysis shows a count of 20 genes (24 probe sets) with GO (biological process) annotations of apoptosis or programmed cell death (p-value = 0.02). Genes in this functional group included apoptosis-inducing factor, oncostatin M (*Osm*), and *Scotin*, and genes involved in cell motility and morphogenesis. Another functional group identified by DAVID (p-value = 0.02) included 4 genes (5 probe sets), *Per1*, *Per2*, *Pde8b*, and *Hif1a*, with a common GO (biological process) annotation: two-component signal transduction system (phospho-relay). Interestingly, these genes have homology to the *Drosophila *PAS/PAC genes thought to be involved in a two component signaling cascade. These molecules are largely attributed to signaling and development in neuronal systems and circadian rhythms, and have not been associated with DC maturation previously [70-72]. This novel observation bears further investigation in relation to DC maturation.

In the clusters described so far, two genes (*Akt3 *and *Hif1a*) have appeared in two separate clusters. The reason for this is that both of these transcripts are targeted by two different probe sets. The *Akt3 *transcript is targeted by 1460307_at (cluster 6) and 1422078_at (cluster 3). Both of these probe set expression profiles peak around 12 hours even though they were placed in different phases, which indicates that this gene remains expressed between 6 and 24 hours. The *Hif1a *transcript is targeted by 1416035_at (cluster 20) and 1427418_a_at (cluster 1). Both of these transcripts were placed in consecutive phases, indicating that *Hif1a *is probably up-regulated at both 12 and 24 hours.

### The dendritic cell maturation "program": temporal patterns of down-regulated gene expression

Over the course of 24 hours the up-regulated program induced by poly(I:C) contained many of the known and well characterized processes induced during DC maturation, as well as several novel observations. However, we were surprised to find that the majority of the overall gene expression patterns in the 2-fold filtered data set exhibited down-regulation (Figure 6d), where 29 of the 42 consensus clusters were down-regulated at some point during the process. Also, it is of interest that the general magnitude of down-regulation is about a 3- to 8-fold (SLR of -1.5 to -3) decrease in expression, with only one cluster exhibiting an expression decrease of 32-fold or more (cluster 8 with SLR of -5). Among the down-regulated gene clusters, none showed consistent repression across all time points. Instead, the pattern in these clusters was waves of down-regulation, peaking early-to-mid, mid, mid-to-late, or late in the DC maturation process (Figure 4). No cluster exhibited significant down-regulation at the earliest analyzed time point of 3 hours.

Unlike the up-regulated clusters, there were very few genes associated with immune response, inflammation, or anti-viral response in the down-regulated clusters. Most of the genes participating in the waves of down-regulation were associated with biosynthetic processes such as transcription, translation and protein synthesis, as well as metabolism. There were even housekeeping genes needed for the maintenance of cellular integrity and metabolism included in these down-regulated clusters. The down-regulation followed a specific time-ordered series of events (Figure 4): transcriptional regulation (early-mid), protein-making machinery and ribosomal processing (mid), translation and metabolic processes (mid-late), and transcription, RNA processing and components of organelles (late).

The first set of genes to be down-regulated were those found in cluster 14, which was observed to be down-regulated at the early-mid phase of 6 to 12 hours (Figure 4, early-mid phase). Three annotation groups were identified by DAVID as being significant: zinc fingers (p-value = 0.01), zinc or cation binding (p-value = 0.04), and nuclear localization (p-value = 0.02). This observation suggests that many genes in this cluster are involved in transcriptional regulation, including *Zfp472 *[73], *Zdhhc14 *[74], *Zfyve21 *and *Statip1*, an activator of transcription. Clearly, transcriptional programming is important in this early-mid phase of DC maturation. Analysis of the temporal program of DC maturation identifies some genes involved in transcription that are down-regulated and others that are up-regulated (see Cluster 3) at this early-mid phase. It is likely that this transcriptional regulation sets the stage for the subsequent maturation process.

Many more clusters and genes were down-regulated during the subsequent phases, mid, mid-late, and late, of the DC maturation process (Figure 4). In the mid-phase, which shows down-regulation of protein-making machinery and ribosomal processing, clusters 2, 7, 8, 12, 16, 19, 31, and 35 contain a number of known genes, including tRNA synthetases and ribosomal proteins (*Yars2*, *Nars2*, *Qrsl1*, *Rps3 *in cluster 2, and *Vars2 *in cluster 8).

DAVID analysis of the mid-late stage shows focus on translation and metabolic machinery. These genes include eukaryotic translation initiation factor 3, subunit 1 alpha (*Eif3s1*) in cluster 24. Additionally, several carbohydrate metabolism genes are down-regulated: ADP-dependent glucokinase (*Adpgk*) in cluster 9 and chondroitin synthase 1 (*Chsy1*) in cluster 17.

Late stage down-regulated genes are significantly annotated with functions related to transcription, RNA processing, pro-survival genes, and components of cellular organelles. A few interferon-related genes are down regulated at this stage: interferon, alpha-inducible protein 27 (*Ifi27*), interleukin 1 beta (*Il1b*), and interferon induced transmembrane protein 3 (*Ifitm3*). Additionally, in this late phase, we observed down-regulation of pro-survival genes, suggesting that apoptosis might be a next stage event (Figure 4, late phase). In general, the down-regulation of this large number of genes across the entire DC maturation process has not been widely reported and paves the way for analysis of the complete transcriptional regulatory network, both up- and down-regulation, for DC maturation.

## Conclusions

The overall goal of this work was to identify the reproducible dynamic features of the DC maturation process induced by poly(I:C) over a period of 24 hours. The integration of replicate data sets into the feature selection filter and consensus clustering analysis identifies genes and gene clusters that show a high level of consistency across replicate experiments. Using one filtering strategy without the other may have its advantages; however, using both together, as done here, allows us to have confidence that the resulting data and biological conclusions are consistent in both scale and pattern across the temporal profile and, thus, represent consistent features of the DC maturation process.

Known and novel biological features were observed from applying this novel filtering strategy. The gene expression patterns observed over the 24 hour time course included many of the expected profiles such as up-regulation in immune response genes. The data also revealed several novel genes that have not been previously associated with the process of DC maturation. We were surprised to find that the down-regulated transcriptional program contained the majority of gene expression patterns and emerged as the dominant feature of the DC maturation process. This program included many genes involved in protein synthesis, metabolism, and housekeeping genes needed for maintenance of cellular integrity and metabolism. The temporal dynamics of these important processes indicate that DC are preparing for cell death.

Our long term objective is to understand the molecular mechanisms underlying the temporal process of DC maturation. Using this global analysis of DC maturation as a guide for future research we can now begin to delve deeper into the finer details of the transcriptional programs that are consistently part of DC maturation induced by poly(I:C), and to compare those programs to those induced by other biological perturbants that stimulate DC maturation. The feature selection method developed here is not limited to DC maturation, time course experiments, or microarray data. The main ideas behind this method can be adapted to other large scale data sets, particularly those which include replicates, to identify consistent features of other biological systems. Consensus clustering can also be used to cluster multiple condition experiments, such as tissue comparisons, which would aid in the identification of groups of genes that consistently respond together in all conditions. Obtaining reproducible results is an important aspect of biological research, and has been the driving force behind the development of the filtering strategy presented here. The results obtained from the application of these methods are the first steps toward understanding the interlaced transcriptional programs that are consistently part of the DC maturation process.

## Methods

### Generation of DC

Bone marrow was harvested from the femurs and tibias of 3-4 month old female C57BL/6 mice (the bone marrow of one mouse was used to initiate the cell culture and generate all of the DC for one experimental replicate). Following lysis of red blood cells, cells were cultured in 20 ng/ml GM-CSF (Biosource) for 6 days [75]. At this point, the cells were typically 85% CD11c+ or higher and had low levels of expression of the DC maturation markers, CD80, CD86, CD40, and MHC class II. To select for only DC, they were purified by CD11c positive selection using the Miltenyi system, according to manufacturer's instructions (Miltenyi). Following purification, the cells were over 97% CD11c+.

### Time course of DC maturation induced by poly(I:C)

Day 6 DC were plated in 6 well plates (1 × 10^7^/well in 5 ml) and treated with 20 μg/ml poly(I:C) (InvivoGen). Following 1, 3, 6, 12, or 24 hours of treatment, the cells were treated with RNAlater (Ambion). RNA was then isolated using the RNAqueous kit (Ambion) according to manufacturer's instructions. Microarray hybridization and preliminary analysis (normalization) was performed by the Wake Forest University microarray core facility using Affymetrix Mouse 430 2.0 oligonucleotide chips.

### Replicate Experiments

Two biological replicate experiments were performed several months apart where each adhered to the above described methods. Microarray hybridization and data normalization was performed independently on each replicate experiment by the array facility; these replicate experiments are referred to as experiments 1 and 2 where experiment 1 was performed first followed by experiment 2.

### Data Pre-processing

All experimental data was normalized according to Affymetrix QC software [76] using MAS5.0 [77] and the standard GCOS settings by the Wake Forest University microarray core facility. Reported data included the change p-value and signal log_2 _ratio (SLR) for each gene. The change p-value was calculated using Wilcoxon's Signed Rank test, a non-parametric alternative to the standard student's t-test [76,78]. A Change (or comparative) call is made based on user defined cut-offs of change p-value, indicating a change in transcript level between a control array and an experiment array, while not necessarily indicating a statistically significant change in expression level. The SLR was calculated using the one-step Tukey's biweight method [76,79] and is a measure of the magnitude and direction of change for each gene on an array compared to the baseline array (control/time zero). The SLR is the fold change on a log_2 _scale [76]; both of these measures of gene expression are used throughout this report.

### Identification of significant differentially expressed genes

Identification of significant differentially expressed genes was performed on each replicate experiment independently, and was carried out in two stages, as outlined in Figure 1A: Step 1. A gene was determined to have a detectible change in transcript level (step 1a) if, at any one time point, the change p-value met the Affymetrix recommended p-value cutoffs of >= 0.998 or <= 0.002 [76], which represents a detectible decrease or increase from the baseline array (time zero), respectively. Additionally, a gene was determined to have significant differential expression (step 1b) if the absolute value of the SLR was >= 1.0 (equivalent to a 2-fold change up or down) at the same time point identified in step 1a. This 2-fold change was chosen based on the current literature standard for differential expression and the knowledge that the MAS5.0 algorithm has been shown to introduce noise into the data when gene expression is low [80-82]. Other normalization methods, such as RMA, have been shown to be more reliable in detecting low gene expression [81]; however, our feature selection method should compensate for this effect by requiring consistency in replicate data and filtering out the noisy data. A more stringent cutoff with an absolute SLR value >= 2.0 (equivalent to a 4-fold change up or down) was also applied to obtain a more focused view of the genes that were highly affected during DC maturation induced by poly(I:C).

### Identification of consistently expressed genes

The identification of consistently expressed genes was carried out in three stages as outlined in Figure 1A: Step 2. A common list of significant differentially expressed genes above detection threshold (obtained from Step 1) was identified in step 2a by taking the intersection of the two independently filtered lists (one from each replicate experiment) at each fold change filtering level. The application of step 2a reduced the four gene lists from Step 1 to two lists that were common to both experiments: one list for the 2-fold filtered and one for the 4-fold filtered data.

Prior to performing steps 2b and 2c on individual gene profiles, Pearson's correlation coefficient (PCC) was calculated between each replicate array using the common gene lists obtained in step 2a for each time point. This was done to ensure that one time point (1, 3, 6, 12 or 24 hours) did not exhibit a large variation between experiments, because subsequent calculations would otherwise be affected along with the consensus clustering analysis (see Additional file 8 for more details). Thus, any time point that did not return a large positive correlation (greater than 0.5) between replicate arrays was excluded from further calculations. The results from this analysis found that the PCC scores for all but the 1 hour arrays were greater than 0.80, indicating reasonable consistency between experiments. The correlation for the 1 hour array, however, was less than 0.40 which indicated poor consistency between experiments; thus this time point was not used in subsequent analysis steps.

Next, steps 2b and 2c were executed by calculating Euclidean distance (ED) and PCC, respectively, for all genes in the two lists obtained from step 2a using the expression profiles from each replicate experiment, where each expression profile consisted of the 3, 6, 12 and 24 hour time points. A gene was considered to have consistently responded to stimuli if the ED between replicate profiles was <= 1.4 (0.0 indicates identity), and the PCC between replicate profiles was >= 0.76 (1.0 indicates identity). Cutoff criteria were determined by calculating the average ED and PCC score for each of the lists of genes obtained from step 2a; thus there were two ED averages and two PCC averages. The most stringent individual cutoff values for ED and PCC were chosen as the consistency filtering criteria for both lists of genes.

ED and PCC are the most influential criteria in this method and could actually be done before filtering for differential expression, resulting in similar gene lists. Here, differential expression filtering was done first to reduce the amount of noise introduced by large numbers of low gene expression so a more accurate threshold for the ED and PCC criteria could be obtained for this data set. Relaxing the PCC and/or ED criteria would introduce genes that were not as consistent across replicates. This in turn would affect the consensus clustering phase.

### Clustering Analysis

Clustering analysis was performed in two stages as outlined in Figure 1A: Step 3 on the gene lists obtained from the feature selection phase. The FOM algorithm (step 3a) was implemented in MATLAB (The MathWorks, Inc.) following the specifications described in Yeung *et al *[43]. An in-house MATLAB program was developed to compare the clustering solutions of MATLAB's k-means and hierarchical agglomerative clustering (HAC) algorithms using the FOM. Briefly, the program clusters the input data using k-means and HAC into a user-specified number of clusters to generate a clustering solution for each algorithm. The FOM score is then calculated for the clustering solutions generated by both algorithms. This process is repeated for a range of increasing cluster numbers, and then the FOM score for each repetition is saved to a file and plotted. The optimal clustering algorithm and the ideal number of clusters are then determined using the set of calculated FOM scores for both clustering algorithms. Euclidean distance was chosen as the similarity measure as the FOM calculation is implicitly biased toward ED, thus would produce inaccurate results if used with any other measure [42]. The analysis was run using the following series of cluster numbers: 2, 4, 6, 8, 10, 12, 14, 16, 18, 20, 22, 24, 26, 28 and 30. The clustering algorithm that yielded the lowest average FOM score over the entire analysis was considered to be optimal [42,83].

After identifying the optimal clustering algorithm, the FOM scores were used to identify the ideal number of clusters for each data set [83]. An in-house algorithm was implemented to calculate the number of clusters where the FOM score is not significantly improved. Briefly, this algorithm calculates the difference in the FOM score for each cluster number range. This was done for all cluster number ranges and the standard deviation of these differences was calculated. The cluster number range that had the smallest difference which was greater than or equal to one standard deviation was chosen as the optimal range for the ideal number of clusters. The maximum value within the optimal range was chosen as the number of clusters into which to break each data set.

The analysis results from step 3a were used as input parameters for the consensus clustering in step 3b. The generation of consensus clusters is performed in three sub-steps: i) all experiment data for one of the gene lists obtained in Step 2 is independently clustered repeatedly to obtain *x *number of clustering solutions each containing *y *clusters (where *y *is the optimal number of clusters determined by FOM); ii) a consensus of all *x *clustering solution is taken to identify genes that are consistently clustered together to form robust (consensus) clusters; iii) a heat map of each consensus cluster is generated using the Clustergram function provided by MATLAB's Bioinformatics Toolbox. This process is then repeated for each gene list obtained in Step 2.

Each replicate data set was clustered 5 times using the k-means algorithm with ED as the similarity measure (10 clustering solutions for each gene list). Choosing a larger or smaller number of k-mean repetitions would have noticeable effects on the resultant clusters. Less repetition would produce fewer consensus clusters with more genes per cluster on average. Likewise, more repetitions would produce more clusters that were smaller in size on average, including a larger number of singleton clusters (data not shown). Based on the familiarity with the data and several trial runs with differing numbers of k-mean repetitions, the number of repetitions was chosen to produce medium sized, stable clusters for analysis.

These clustering solutions were input into an in-house MATLAB program designed to identify all consensus clusters. This program follows a similar algorithm to Monti *et al *[32] and Swift *et al *[33] with minor differences. Briefly, each of the *n *genes in the input data set were paired with every other gene in that data set which created an *n *× *n *matrix using the gene IDs as the row and column labels. The algorithm examined each of the *x *clustering solutions one at a time; every time a pair of genes was found in the same cluster a count for that gene pair was incremented by one in the corresponding matrix location. If a pair of genes was found in the same cluster in all *x *clustering solutions, then the count for that pair was equal to *x*, otherwise the count was less than *x*. Consensus clusters were extracted by examining each gene, *r*, in the data set. A group of genes *c *that was found in the same cluster as gene *r *for all clustering solutions was identified as a consensus cluster.

After the consensus clusters were identified, MATLAB's Clustergram function was used to generate heat maps for visualization. This function hierarchically clustered each consensus cluster, using ED as the similarity measure, to organize the genes according to greatest similarity in expression level.

It is worth mentioning that the resultant consensus clusters can potentially be affected by the normalization method chosen during the pre-processing stage of analysis. It has been shown that normalization methods such as RMA and GCRMA, while able to detect lower expression changes more reliably [80-82], add a significant number of correlative artifacts to the data [84]. This would affect the consensus clustering where genes would appear to cluster more robustly than they would otherwise. Thus, it is important to consider which normalization procedure to use depending on the type of subsequent analyses that are going to be performed.

### DAVID Analysis

The functional annotation clustering tool provided by DAVID [52,85,86] was used to analyze a subset of the 2-fold consensus clusters. This subset was chosen to be the largest 42 consensus clusters (those with 10 genes or more; the top 76% of genes) for the 2-fold filtered gene list. This cluster size cutoff was chosen because any cluster smaller than 10 genes returned few (if any) significant results from DAVID. The list of Affymetrix IDs for each consensus cluster was input into DAVID as separate lists. For each cluster list the background data set was chosen to be the Affymetrix Mouse430_2 chip, and only those genes associated with *Mus musculus *were selected for analysis by DAVID. The Gene Ontology (GO) search terms were changed from the default of ALL to levels 3, 4 and 5 for the biological process, cellular component and molecular function. The terms associated with each cluster's list of genes were then clustered using the functional annotation clustering tool with the classification stringency set to the default high setting. Functional groups that had an enrichment score greater than 1.3 (p-value of 0.05 or less) were considered significant and analyzed further.

## Abbreviations

ED: Euclidean distance; PCC: Pearson's correlation coefficient; DC: dendritic cells; BMDC: bone marrow-derived dendritic cells; FOM: figure of merit; SLR: signal log ratio; HAC: hierarchical agglomerative clustering; GO: Gene Ontology.

## Authors' contributions

JSF and EMH conceived of the analysis. EMH performed the microarray experiments and provided expert input into biological interpretation of results. ALO created the analysis (including scripts and programs) described in this manuscript and performed all analyses on the experimental data. JSF worked closely with ALO to provide feedback at all stages of development of the filtering strategy and to outline the manuscript. XL contributed expertise to the statistical portions of this manuscript. ALO drafted the first version of the manuscript and all authors were involved in significant editing and re-writing of the manuscript. All authors read and approved the final manuscript prior to submission.

## Supplementary Material

Additional file 1 **ED and PCC filtering example**. Examples of why both ED and PCC criteria are important to identifying consistent gene expression profiles. Click here for file

Additional file 2 **Average expression profile example**. Demonstration of how the average expression profile does not always represent either of the original profiles. Click here for file

Additional file 3 **Consensus clusters exhibit different expression patterns in each replicate**. Figure showing one consensus cluster that has a slightly different expression pattern in replicate experiments, and an explanation of the potential this has for the proposed method. Click here for file

Additional file 4 **Supplementary tables 1 and 2**. MS Excel workbook containing 2 tables of SLR expression values and cluster assignments for the 2-fold and 4-fold consensus clusters discussed in this manuscript. Click here for file

Additional file 5 **Flow cytometry results**. Bone marrow-derived DC up regulate cell surface CD86 in response to treatment with poly(I:C). Click here for file

Additional file 6 **Validation of microarray data**. Correlation coefficients and scatter plots generated by comparing microarray data from this study to a similar study done by Amit *et al*. Click here for file

Additional file 7 **Annotation comparison to published results**. Summary of a DAVID annotation analysis to compare annotation terms present in each temporal phase of response in the 4-fold filtered data set to those common response genes reported in Huang *et al*. Click here for file

Additional file 8 **Justification for removing the 1 hour time point**. Discussion of a parallel analysis done using the 1 hour time point for filtering and consensus clustering, and the impact it had on the results. Click here for file

## References

[B1] Wilkes T Laux H Foy CA Microarray data quality-review of current developments Omics 2007 11 1 13 10.1089/omi.2006.0001 17411392

[B2] Dopazo J Zanders E Dragoni I Amphlett G Falciani F Methods and approaches in the analysis of gene expression data J Immunol Methods 2001 250 93 112 10.1016/S0022-1759(01)00307-6 11251224

[B3] Banchereau J Steinman RM Dendritic cells and the control of immunity Nature 1998 392 245 52 10.1038/32588 9521319

[B4] Guermonprez P Valladeau J Zitvogel L Thery C Amigorena S Antigen presentation and T cell stimulation by dendritic cells Annu Rev Immunol 2002 20 621 67 10.1146/annurev.immunol.20.100301.064828 11861614

[B5] Huang Q Liu D Majewski P Schulte LC Korn JM Young RA Lander ES Hacohen N The plasticity of dendritic cell responses to pathogens and their components Science 2001 294 870 5 10.1126/science.294.5543.870 11679675

[B6] Steinman RM Pack M Inaba K Dendritic cell development and maturation Adv Exp Med Biol 1997 417 1 6 9286329 10.1007/978-1-4757-9966-8_1

[B7] Trombetta ES Ebersold M Garrett W Pypaert M Mellman I Activation of lysosomal function during dendritic cell maturation Science 2003 299 1400 3 10.1126/science.1080106 12610307

[B8] Turley SJ Inaba K Garrett WS Ebersold M Unternaehrer J Steinman RM Mellman I Transport of peptide-MHC class II complexes in developing dendritic cells Science 2000 288 522 7 10.1126/science.288.5465.522 10775112

[B9] Pierre P Shachar I Matza D Gatti E Flavell RA Mellman I Invariant chain controls H2-M proteolysis in mouse splenocytes and dendritic cells J Exp Med 2000 191 1057 62 10.1084/jem.191.6.1057 10727467 PMC2193111

[B10] Delamarre L Pack M Chang H Mellman I Trombetta ES Differential lysosomal proteolysis in antigen-presenting cells determines antigen fate Science 2005 307 1630 4 10.1126/science.1108003 15761154

[B11] Chow A Toomre D Garrett W Mellman I Dendritic cell maturation triggers retrograde MHC class II transport from lysosomes to the plasma membrane Nature 2002 418 988 94 10.1038/nature01006 12198549

[B12] Garrett WS Chen LM Kroschewski R Ebersold M Turley S Trombetta S Galan JE Mellman I Developmental control of endocytosis in dendritic cells by Cdc42 Cell 2000 102 325 34 10.1016/S0092-8674(00)00038-6 10975523

[B13] Rescigno M Martino M Sutherland CL Gold MR Ricciardi-Castagnoli P Dendritic cell survival and maturation are regulated by different signaling pathways J Exp Med 1998 188 2175 80 10.1084/jem.188.11.2175 9841930 PMC2212396

[B14] Bertho N Blancheteau VM Setterblad N Laupeze B Lord JM Drenou B Amiot L Charron DJ Fauchet R Mooney N MHC class II-mediated apoptosis of mature dendritic cells proceeds by activation of the protein kinase C-delta isoenzyme Int Immunol 2002 14 935 42 10.1093/intimm/dxf058 12147630

[B15] Kim JH Chen J Majumder N Lin H Falo LD Jr You Z 'Survival gene' Bcl-xl potentiates DNA-raised antitumor immunity Gene Ther 2005 12 1517 25 10.1038/sj.gt.3302584 16052205

[B16] Severa M Remoli ME Giacomini E Ragimbeau J Lande R Uze G Pellegrini S Coccia EM Differential responsiveness to IFN-alpha and IFN-beta of human mature DC through modulation of IFNAR expression J Leukoc Biol 2006 79 1286 94 10.1189/jlb.1205742 16624932

[B17] Granucci F Vizzardelli C Pavelka N Feau S Persico M Virzi E Rescigno M Moro G Ricciardi-Castagnoli P Inducible IL-2 production by dendritic cells revealed by global gene expression analysis Nat Immunol 2001 2 882 8 10.1038/ni0901-882 11526406

[B18] McIlroy D Tanguy-Royer S Le Meur N Guisle I Royer PJ Leger J Meflah K Gregoire M Profiling dendritic cell maturation with dedicated microarrays J Leukoc Biol 2005 78 794 803 10.1189/jlb.0105029 15961579

[B19] Schoeters E Nuijten JM Van Den Heuvel RL Nelissen I Witters H Schoeters GE Van Tendeloo VF Berneman ZN Verheyen GR Gene expression signatures in CD34+-progenitor-derived dendritic cells exposed to the chemical contact allergen nickel sulfate Toxicol Appl Pharmacol 2006 216 131 49 10.1016/j.taap.2006.04.009 16780908

[B20] Vizzardelli C Pavelka N Luchini A Zanoni I Bendickson L Pelizzola M Beretta O Foti M Granucci F Nilsen-Hamilton M Effects of dexamethazone on LPS-induced activationand migration of mouse dendritic cells revealed by a genome-wide transcriptional analysis Eur J Immunol 2006 36 1504 15 10.1002/eji.200535488 16708398

[B21] Zilliox MJ Parmigiani G Griffin DE Gene expression patterns in dendritic cells infected with measles virus compared with other pathogens Proc Natl Acad Sci USA 2006 103 3363 8 10.1073/pnas.0511345103 16492729 PMC1413941

[B22] Coombes KR Highsmith WE Krogmann TA Baggerly KA Stivers DN Abruzzo LV Identifying and quantifying sources of variation in microarray data using high-density cDNA membrane arrays J Comput Biol 2002 9 655 69 10.1089/106652702760277372 12323099

[B23] Kerr MK Martin M Churchill GA Analysis of variance for gene expression microarray data J Comput Biol 2000 7 819 37 10.1089/10665270050514954 11382364

[B24] Wu B Differential gene expression detection using penalized linear regression models: the improved SAM statistics Bioinformatics 2005 21 1565 71 10.1093/bioinformatics/bti217 15598833

[B25] Tusher VG Tibshirani R Chu G Significance analysis of microarrays applied to the ionizing radiation response Proc Natl Acad Sci USA 2001 98 5116 21 10.1073/pnas.091062498 11309499 PMC33173

[B26] Jeffery IB Higgins DG Culhane AC Comparison and evaluation of methods for generating differentially expressed gene lists from microarray data BMC Bioinformatics 2006 7 359 10.1186/1471-2105-7-359 16872483 PMC1544358

[B27] Liang Y Tayo B Cai X Kelemen A Differential and trajectory methods for time course gene expression data Bioinformatics 2005 21 3009 16 10.1093/bioinformatics/bti465 15886280 PMC2574001

[B28] Draghici S Kulaeva O Hoff B Petrov A Shams S Tainsky MA Noise sampling method: an ANOVA approach allowing robust selection of differentially regulated genes measured by DNA microarrays Bioinformatics 2003 19 1348 59 10.1093/bioinformatics/btg165 12874046

[B29] Jiang D Tang C Zhang A Cluster Analysis for Gene Expression Data: A Survey IEEE Transactions on Knowledge and Data Engineering 2004 16 1370 1386 10.1109/TKDE.2004.68

[B30] Eisen MB Spellman PT Brown PO Botstein D Cluster analysis and display of genome-wide expression patterns Proc Natl Acad Sci USA 1998 95 14863 8 10.1073/pnas.95.25.14863 9843981 PMC24541

[B31] Tavazoie S Hughes JD Campbell MJ Cho RJ Church GM Systematic determination of genetic network architecture Nat Genet 1999 22 281 5 10.1038/10343 10391217

[B32] Monti S Tamayo P Mesirov J Golub T Consensus Clustering: A resampling-based method for class discovery and visualization of gene expression microarray data Machine Learning 2003 52 91 118 10.1023/A:1023949509487

[B33] Swift S Tucker A Vinciotti V Martin N Orengo C Liu X Kellam P Consensus clustering and functional interpretation of gene-expression data Genome Biol 2004 5 R94 10.1186/gb-2004-5-11-r94 15535870 PMC545785

[B34] Yeung KY Medvedovic M Bumgarner RE Clustering gene-expression data with repeated measurements Genome Biol 2003 4 R34 10.1186/gb-2003-4-5-r34 12734014 PMC156590

[B35] Kim J Kim JH Difference-based clustering of short time-course microarray data with replicates BMC Bioinformatics 2007 8 253 10.1186/1471-2105-8-253 17629922 PMC1952071

[B36] Tjaden B An approach for clustering gene expression data with error information BMC Bioinformatics 2006 7 17 10.1186/1471-2105-7-17 16409635 PMC1360687

[B37] Yao J Chang C Salmi ML Hung YS Loraine A Roux SJ Genome-scale cluster analysis of replicated microarrays using shrinkage correlation coefficient BMC Bioinformatics 2008 9 288 10.1186/1471-2105-9-288 18564431 PMC2459189

[B38] Luan Y Li H Clustering of time-course gene expression data using a mixed-effects model with B-splines Bioinformatics 2003 19 474 82 10.1093/bioinformatics/btg014 12611802

[B39] Ma P Castillo-Davis CI Zhong W Liu JS A data-driven clustering method for time course gene expression data Nucleic Acids Res 2006 34 1261 9 10.1093/nar/gkl013 16510852 PMC1388097

[B40] Storey JD Xiao W Leek JT Tompkins RG Davis RW Significance analysis of time course microarray experiments Proc Natl Acad Sci USA 2005 102 12837 42 10.1073/pnas.0504609102 16141318 PMC1201697

[B41] Kung C Kenski DM Dickerson SH Howson RW Kuyper LF Madhani HD Shokat KM Chemical genomic profiling to identify intracellular targets of a multiplex kinase inhibitor Proc Natl Acad Sci USA 2005 102 3587 92 10.1073/pnas.0407170102 15738404 PMC552777

[B42] Olex AL John DJ Hiltbold EM Fetrow JS Additional limitations of the clustering validation method figure of merit45th ACM Southeast Annual Conference 2007 Winston-Salem, NC 238 243 full_text

[B43] Yeung KY Haynor DR Ruzzo WL Validating clustering for gene expression data Bioinformatics 2001 17 309 18 10.1093/bioinformatics/17.4.309 11301299

[B44] Handl J Knowles J Kell DB Computational cluster validation in post-genomic data analysis Bioinformatics 2005 21 3201 12 10.1093/bioinformatics/bti517 15914541

[B45] Giancarlo R Scaturro D Utro F Computational cluster validation for microarray data analysis: experimental assessment of Clest, Consensus Clustering, Figure of Merit, Gap Statistics and Model Explorer BMC Bioinformatics 2008 9 462 10.1186/1471-2105-9-462 18959783 PMC2657801

[B46] Dudoit S Fridlyand J A prediction-based resampling method for estimating the number of clusters in a dataset Genome Biol 2002 3 RESEARCH0036 10.1186/gb-2002-3-7-research0036 12184810 PMC126241

[B47] Tibshirani R Walther G Hastie T Estimating the Number of Clusters in a Dataset via the Gap Statistics Journal Royal Statistical Society B 2001 2 411 423 10.1111/1467-9868.00293

[B48] Ben-Hur A Elisseeff A Guyon I A stability based method for discovering structure in clustered data Pac Symp Biocomput 2002 6 17 11928511

[B49] Amit I Garber M Chevrier N Leite AP Donner Y Eisenhaure T Guttman M Grenier JK Li W Zuk O Unbiased reconstruction of a mammalian transcriptional network mediating pathogen responses Science 2009 326 257 63 10.1126/science.1179050 19729616 PMC2879337

[B50] Honda K Sakaguchi S Nakajima C Watanabe A Yanai H Matsumoto M Ohteki T Kaisho T Takaoka A Akira S Selective contribution of IFN-alpha/beta signaling to the maturation of dendritic cells induced by double-stranded RNA or viral infection Proc Natl Acad Sci USA 2003 100 10872 7 10.1073/pnas.1934678100 12960379 PMC196895

[B51] Lore K Betts MR Brenchley JM Kuruppu J Khojasteh S Perfetto S Roederer M Seder RA Koup RA Toll-like receptor ligands modulate dendritic cells to augment cytomegalovirus- and HIV-1-specific T cell responses J Immunol 2003 171 4320 8 14530357 10.4049/jimmunol.171.8.4320

[B52] Dennis G Jr Sherman BT Hosack DA Yang J Gao W Lane HC Lempicki RA DAVID: Database for Annotation, Visualization, and Integrated Discovery Genome Biol 2003 4 P3 10.1186/gb-2003-4-5-p3 12734009 PMC193660

[B53] Sato M Suemori H Hata N Asagiri M Ogasawara K Nakao K Nakaya T Katsuki M Noguchi S Tanaka N Distinct and essential roles of transcription factors IRF-3 and IRF-7 in response to viruses for IFN-alpha/beta gene induction Immunity 2000 13 539 48 10.1016/S1074-7613(00)00053-4 11070172

[B54] Espert L Degols G Lin YL Vincent T Benkirane M Mechti N Interferon-induced exonuclease ISG20 exhibits an antiviral activity against human immunodeficiency virus type 1 J Gen Virol 2005 86 2221 9 10.1099/vir.0.81074-0 16033969

[B55] Espert L Degols G Gongora C Blondel D Williams BR Silverman RH Mechti N ISG20, a new interferon-induced RNase specific for single-stranded RNA, defines an alternative antiviral pathway against RNA genomic viruses J Biol Chem 2003 278 16151 8 10.1074/jbc.M209628200 12594219

[B56] Bain VG Yoshida EM Kaita KD Swain MG Heathcote EJ Garcia A Moore PA Yu R McHutchison JG Subramanian GM Dynamics of interferon-specific gene expression in peripheral blood of interferon alfa-naive patients with genotype 1 chronic hepatitis C infection treated with albumin-interferon alfa Hepatol Res 2006 35 256 62 10.1016/j.hepres.2006.04.005 16731032

[B57] Sanda C Weitzel P Tsukahara T Schaley J Edenberg HJ Stephens MA McClintick JN Blatt LM Li L Brodsky L Differential gene induction by type I and type II interferons and their combination J Interferon Cytokine Res 2006 26 462 72 10.1089/jir.2006.26.462 16800785

[B58] Megiovanni AM Sanchez F Gluckman JC Rosenzwajg M Double-stranded RNA stimulation or CD40 ligation of monocyte-derived dendritic cells as models to study their activation and maturation process Eur Cytokine Netw 2004 15 126 34 15319172

[B59] Takahashi N Yamada T Narita N Fujieda S Double-stranded RNA induces production of RANTES and IL-8 by human nasal fibroblasts Clin Immunol 2006 118 51 8 10.1016/j.clim.2005.09.001 16253565

[B60] Genin P Algarte M Roof P Lin R Hiscott J Regulation of RANTES chemokine gene expression requires cooperativity between NF-kappa B and IFN-regulatory factor transcription factors J Immunol 2000 164 5352 61 10799898 10.4049/jimmunol.164.10.5352

[B61] Brady G Boggan L Bowie A O'Neill LA Schlafen-1 causes a cell cycle arrest by inhibiting induction of cyclin D1 J Biol Chem 2005 280 30723 34 10.1074/jbc.M500435200 15946944

[B62] Geserick P Kaiser F Klemm U Kaufmann SH Zerrahn J Modulation of T cell development and activation by novel members of the Schlafen (slfn) gene family harbouring an RNA helicase-like motif Int Immunol 2004 16 1535 48 10.1093/intimm/dxh155 15351786

[B63] Schwarz DA Katayama CD Hedrick SM Schlafen, a new family of growth regulatory genes that affect thymocyte development Immunity 1998 9 657 68 10.1016/S1074-7613(00)80663-9 9846487

[B64] Hagnerud S Manna PP Cella M Stenberg A Frazier WA Colonna M Oldenborg PA Deficit of CD47 results in a defect of marginal zone dendritic cells, blunted immune response to particulate antigen and impairment of skin dendritic cell migration J Immunol 2006 176 5772 8 16670282 10.4049/jimmunol.176.10.5772

[B65] Latour S Tanaka H Demeure C Mateo V Rubio M Brown EJ Maliszewski C Lindberg FP Oldenborg A Ullrich A Bidirectional negative regulation of human T and dendritic cells by CD47 and its cognate receptor signal-regulator protein-alpha: down-regulation of IL-12 responsiveness and inhibition of dendritic cell activation J Immunol 2001 167 2547 54 11509594 10.4049/jimmunol.167.5.2547

[B66] Demeure CE Tanaka H Mateo V Rubio M Delespesse G Sarfati M CD47 engagement inhibits cytokine production and maturation of human dendritic cells J Immunol 2000 164 2193 9 10657674 10.4049/jimmunol.164.4.2193

[B67] Gilchrist M Thorsson V Li B Rust AG Korb M Roach JC Kennedy K Hai T Bolouri H Aderem A Systems biology approaches identify ATF3 as a negative regulator of Toll-like receptor 4 Nature 2006 441 173 8 10.1038/nature04768 16688168

[B68] Townsend A Trowsdale J The transporters associated with antigen presentation Semin Cell Biol 1993 4 53 61 10.1006/scel.1993.1007 8453065

[B69] Rufer E Leonhardt RM Knittler MR Molecular architecture of the TAP-associated MHC class I peptide-loading complex J Immunol 2007 179 5717 27 17947644 10.4049/jimmunol.179.9.5717

[B70] Akashi M Ichise T Mamine T Takumi T Molecular mechanism of cell-autonomous circadian gene expression of Period2, a crucial regulator of the mammalian circadian clock Mol Biol Cell 2006 17 555 65 10.1091/mbc.E05-05-0396 16280364 PMC1356568

[B71] Yamamoto T Nakahata Y Soma H Akashi M Mamine T Takumi T Transcriptional oscillation of canonical clock genes in mouse peripheral tissues BMC Mol Biol 2004 5 18 10.1186/1471-2199-5-18 15473909 PMC535906

[B72] Shigeyoshi Y Meyer-Bernstein E Yagita K Fu W Chen Y Takumi T Schotland P Sehgal A Okamura H Restoration of circadian behavioural rhythms in a period null Drosophila mutant (per01) by mammalian period homologues mPer1 and mPer2 Genes Cells 2002 7 163 71 10.1046/j.1356-9597.2001.00503.x 11895480

[B73] Jiang L Tang D Wang K Zhang H Yuan C Duan D Xiao X Functional analysis of a novel KRAB/C2H2 zinc finger protein Mipu1 Biochem Biophys Res Commun 2007 356 829 35 10.1016/j.bbrc.2007.02.138 17397802

[B74] Rinaldi A Kwee I Poretti G Mensah A Pruneri G Capello D Rossi D Zucca E Ponzoni M Catapano C Comparative genome-wide profiling of post-transplant lymphoproliferative disorders and diffuse large B-cell lymphomas Br J Haematol 2006 134 27 36 10.1111/j.1365-2141.2006.06114.x 16803564

[B75] Inaba K Inaba M Romani N Aya H Deguchi M Ikehara S Muramatsu S Steinman RM Generation of large numbers of dendritic cells from mouse bone marrow cultures supplemented with granulocyte/macrophage colony-stimulating factor J Exp Med 1992 176 1693 702 10.1084/jem.176.6.1693 1460426 PMC2119469

[B76] GeneChip Expression Analysis: Data Analysis Fundamentals https://www.affymetrix.com/support/downloads/manuals/data_analysis_fundamentals_manual.pdf

[B77] Pepper SD Saunders EK Edwards LE Wilson CL Miller CJ The utility of MAS5 expression summary and detection call algorithms BMC Bioinformatics 2007 8 273 10.1186/1471-2105-8-273 17663764 PMC1950098

[B78] "Wilcoxon Signed Rank Test" From MathWorld -- A Wolfram Web Resource http://mathworld.wolfram.com/WilcoxonSignedRankTest.html

[B79] "Tukey's Biweight" From MathWorld -- A Wolfram Web Resource http://mathworld.wolfram.com/TukeysBiweight.html

[B80] Irizarry RA Bolstad BM Collin F Cope LM Hobbs B Speed TP Summaries of Affymetrix GeneChip probe level data Nucleic Acids Res 2003 31 e15 10.1093/nar/gng015 12582260 PMC150247

[B81] Millenaar FF Okyere J May ST van Zanten M Voesenek LA Peeters AJ How to decide? Different methods of calculating gene expression from short oligonucleotide array data will give different results BMC Bioinformatics 2006 7 137 10.1186/1471-2105-7-137 16539732 PMC1431565

[B82] Qin LX Beyer RP Hudson FN Linford NJ Morris DE Kerr KF Evaluation of methods for oligonucleotide array data via quantitative real-time PCR BMC Bioinformatics 2006 7 23 10.1186/1471-2105-7-23 16417622 PMC1360686

[B83] Yeung KY Cluster Analysis of Gene Expression Data Dissertation 2001 Seattle, WA: University of Washington

[B84] Lim WK Wang K Lefebvre C Califano A Comparative analysis of microarray normalization procedures: effects on reverse engineering gene networks Bioinformatics 2007 23 i282 8 10.1093/bioinformatics/btm201 17646307

[B85] Huang da W Sherman BT Tan Q Kir J Liu D Bryant D Guo Y Stephens R Baseler MW Lane HC DAVID Bioinformatics Resources: expanded annotation database and novel algorithms to better extract biology from large gene lists Nucleic Acids Res 2007 35 W169 75 10.1093/nar/gkm415 17576678 PMC1933169

[B86] Sherman BT Huang da W Tan Q Guo Y Bour S Liu D Stephens R Baseler MW Lane HC Lempicki RA DAVID Knowledgebase: a gene-centered database integrating heterogeneous gene annotation resources to facilitate high-throughput gene functional analysis BMC Bioinformatics 2007 8 426 10.1186/1471-2105-8-426 17980028 PMC2186358

