# Supplementary material for: Dynamics of dendritic cell maturation are identified through a novel filtering strategy applied to biological time-course microarray replicates
Source: BMC Immunol. 2010 Aug 3;11:41. doi: 10.1186/1471-2172-11-41 (PMC2928180; doi:10.1186/1471-2172-11-41)
Supplement: Additional file 1 — ED and PCC filtering example. Examples of why both ED and PCC criteria are important to identifying consistent gene expression profiles. [file 1471-2172-11-41-S1.PDF]

Additional file 1 - Satisfaction of both ED and PCC criteria returns the most consistent profiles across replicate experiments.

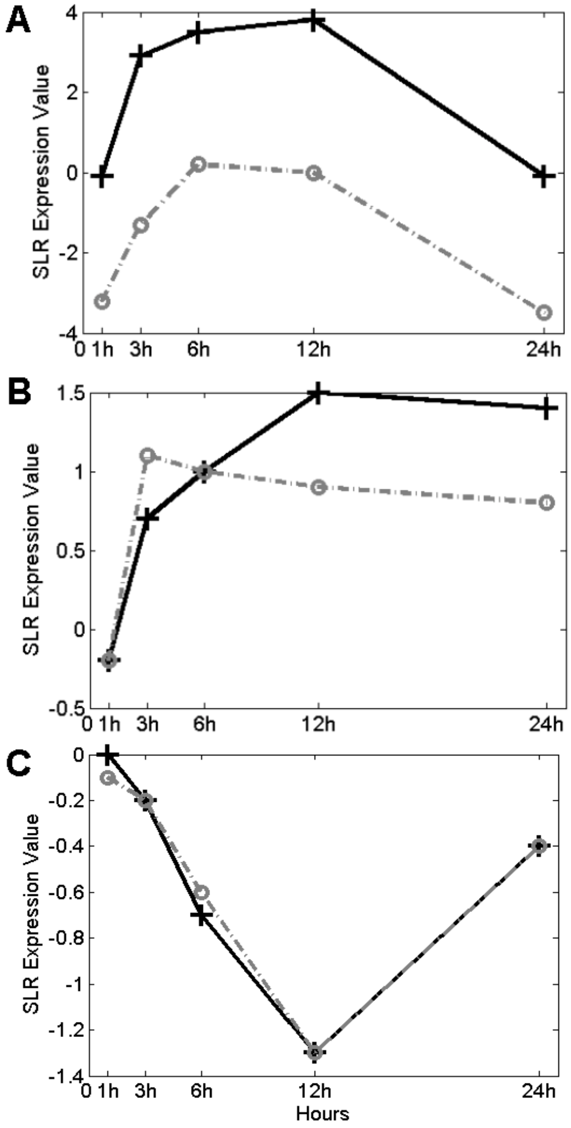

Three probe sets were chosen to demonstrate that both Euclidean distance (ED) and Pearson's correlation coefficient (PCC) are required to identify consistent replicate expression profiles. Replicate profiles with good correlation can still have significant differences in levels of expression (PCC=0.97 and ED=7.4 in Supplementary Figure 1A). The other extreme is that replicate expression profiles can have very similar levels of expression, but exhibit a negative correlation (PCC= -0.91 and ED=0.94 in Supplementary Figure 1B). These examples demonstrate that satisfaction of only the ED or PCC criteria does not guarantee that the levels of expression and profile pattern are the same between experiments. However, replicate profiles exhibiting both similar levels of expression and a strong positive correlation between replicates is an example of an ideal expression profile where both the PCC and ED criteria are met (ED=0.1 and PCC=0.99 in Supplementary Figure 1C). Expression profiles that only satisfied one of the ED and PCC criteria, such as those in (A) and (B), were not included in the final data sets.

Supplementary Figure 1: Key - experiment 1, black solid line; experiment 2, gray dashed line. Affymetrix IDs are 1443693_at (A), 1460205_at (B) and 1417082_at (C).
