# Supplementary material for: Dynamics of dendritic cell maturation are identified through a novel filtering strategy applied to biological time-course microarray replicates
Source: BMC Immunol. 2010 Aug 3;11:41. doi: 10.1186/1471-2172-11-41 (PMC2928180; doi:10.1186/1471-2172-11-41)
Supplement: Additional file 2 — Average expression profile example. Demonstration of how the average expression profile does not always represent either of the original profiles. [file 1471-2172-11-41-S2.PDF]

Additional file 2: The average expression profile between replicate experiments does not always accurately represent the original profiles.

The figure below shows 3 examples of probe sets that had significant change p-values (>= 0.05) for all time points in both replicate experiments, but the average profile looks very different from either of the original profiles. If the filtering process was only based on the change p-value and a fold change of 2.0 (SLR >= 1.0 or <= -1.0) then all three of these average profiles would be included in the significant gene list even though the average profile is a poor representation of what actually was measured.

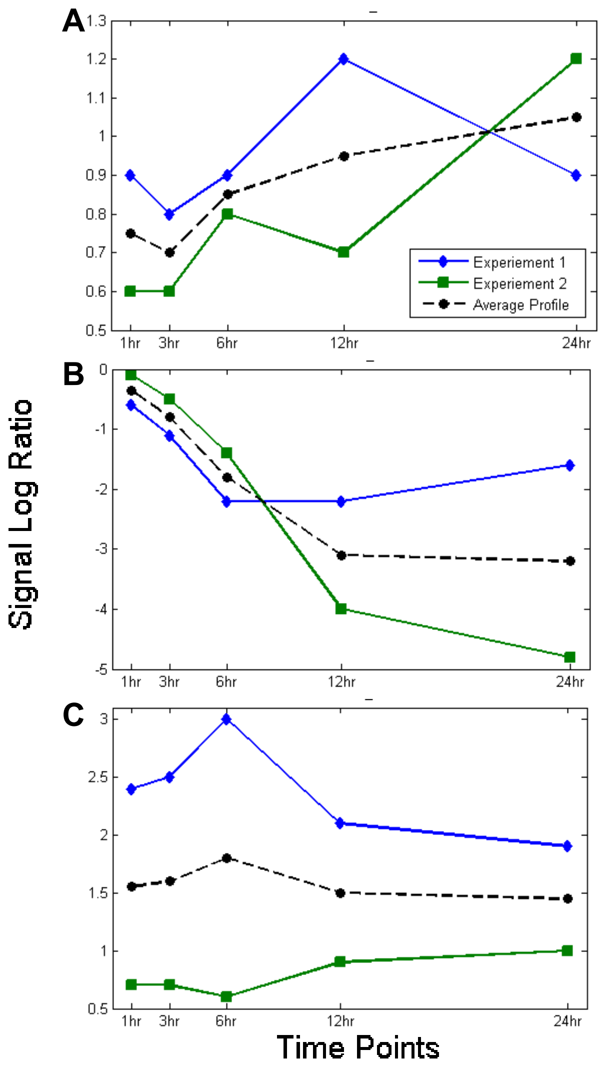

Supplementary Figure 2: Three examples of average profiles that are not similar to either of the original profiles used in the calculation. Each plot represents the profile data from experiment 1 (blue), experiment 2 (green) and the average (black) for a single probe set. Affymetrix probe sets shown are 1423006_at (A), 1423357_at (B) and 1424684_at (C).
