# Supplementary material for: Dynamics of dendritic cell maturation are identified through a novel filtering strategy applied to biological time-course microarray replicates
Source: BMC Immunol. 2010 Aug 3;11:41. doi: 10.1186/1471-2172-11-41 (PMC2928180; doi:10.1186/1471-2172-11-41)
Supplement: Additional file 3 — Consensus clusters exhibit different expression patterns in each replicate. Figure showing one consensus cluster that has a slightly different expression pattern in replicate experiments, and an explanation of the potential this has for the proposed method. [file 1471-2172-11-41-S3.PDF]

Additional file 3: Clustered genes that exhibit slightly different expression patterns between replicates may have some common regulatory mechanism.

To illustrate that consensus clustering has the potential to identify groups of genes that respond to stimuli as a group, but have a different response in each biological replicate experiment, we show two heat maps of a single consensus cluster (cluster 19) generated from experiments 1 and 2 (Supplementary Figure 3). These 5 temporal profiles (5 probe sets representing 4 unique genes) are not perfectly consistent across the experiments—an expected result in biological replicate experiments. In experiment 1 (Supplementary Figure 3, left), all 5 probe sets reach a peak repression at 12 hours whereas in experiment 2 (Supplementary Figure 3, right) the peak occurs at 24 hours. This discrepancy may be a result of experimental variation such as inexact timing of stimulation or harvest; however, consensus clustering identifies a strong relationship between these genes because all 5 expression profiles changed together in the second experiment, suggesting that these 4 genes may have some common regulatory motif or mechanism for future investigation.

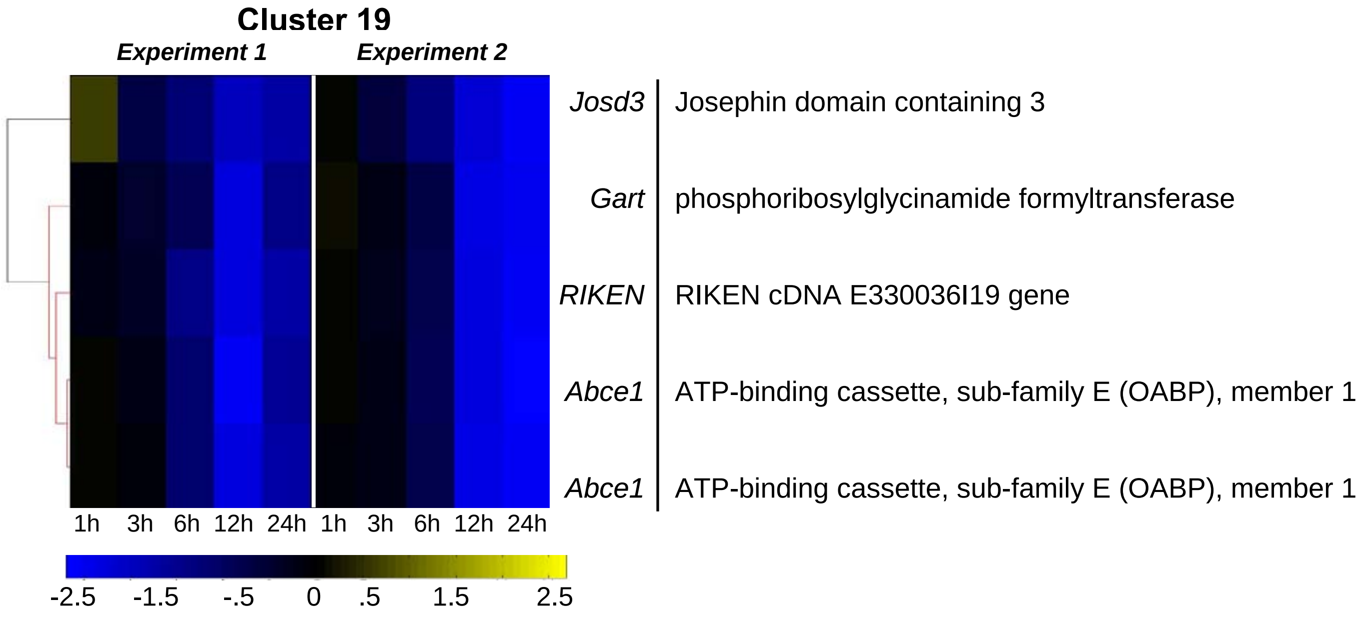

Supplementary Figure 3: A heat map representation of a consensus cluster containing genes that were expressed differently in replicate experiments, but still formed a robust cluster. The color scale is the SLR of each gene's expression (blue = down-regulated, yellow = up-regulated).
