# Supplementary material for: Dynamics of dendritic cell maturation are identified through a novel filtering strategy applied to biological time-course microarray replicates
Source: BMC Immunol. 2010 Aug 3;11:41. doi: 10.1186/1471-2172-11-41 (PMC2928180; doi:10.1186/1471-2172-11-41)
Supplement: Additional file 5 — Flow cytometry results. Bone marrow-derived DC up regulate cell surface CD86 in response to treatment with poly(I:C). [file 1471-2172-11-41-S5.PDF]

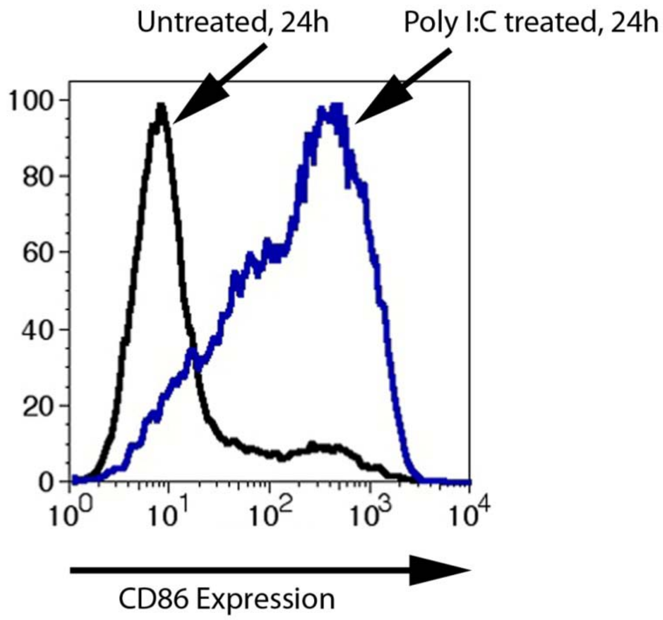

Additional file 5: BMDC up regulation of cell surface CD86 upon Poly I:C treatment.

BMDC were treated with Poly(I:C) (2.5 ug/ml) for 24h or left untreated. Cells were harvested and stained with a fluorescently conjugated antibody against CD86 (clone GL1) and analyzed by flow cytometry. The mean fluorescent intensity of the untreated cells (black line) was 13 and the treated cells (blue line) was 158.
