# Supplementary material for: Dynamics of dendritic cell maturation are identified through a novel filtering strategy applied to biological time-course microarray replicates
Source: BMC Immunol. 2010 Aug 3;11:41. doi: 10.1186/1471-2172-11-41 (PMC2928180; doi:10.1186/1471-2172-11-41)
Supplement: Additional file 6 — Validation of microarray data. Correlation coefficients and scatter plots generated by comparing microarray data from this study to a similar study done by Amit et al. [file 1471-2172-11-41-S6.PDF]

Additional file 6: Microarray data is positively correlated with previously published data.

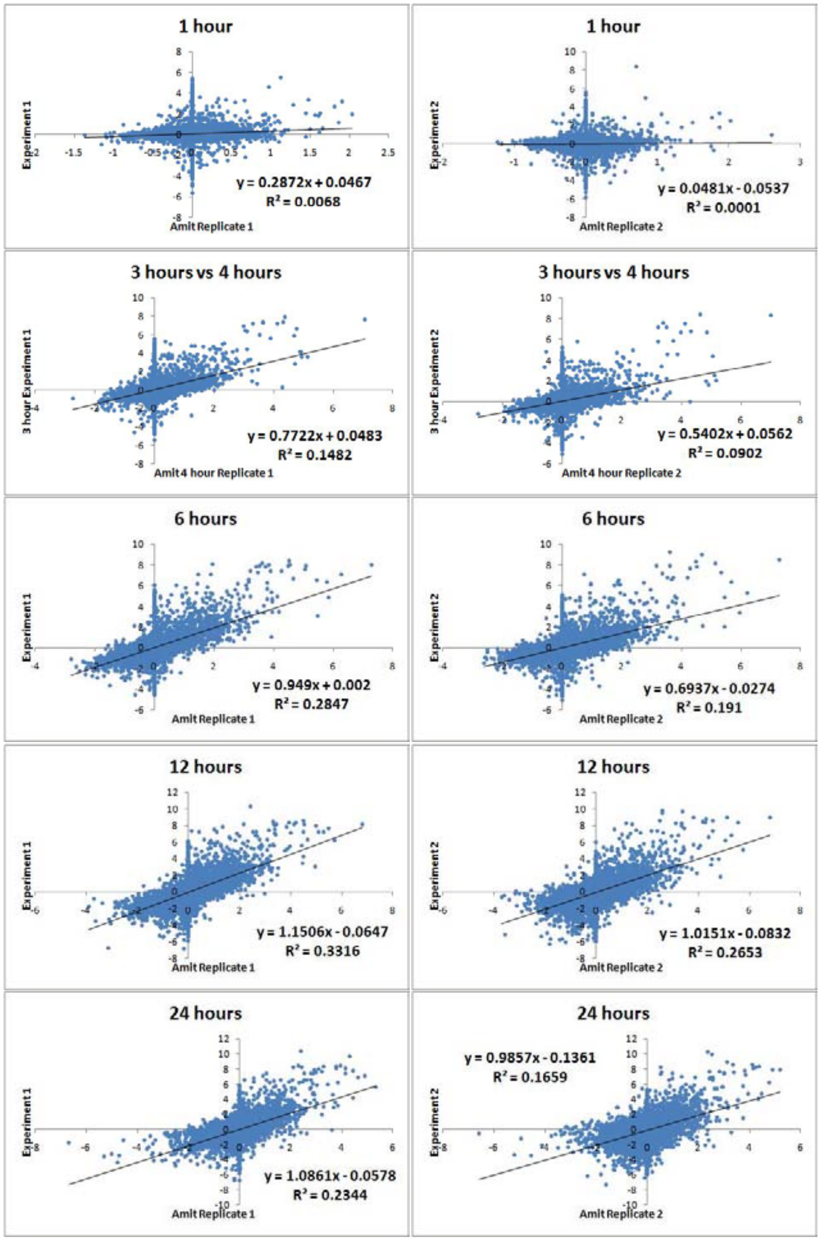

Comparison of microarray data was made to a similar experiment published by Amit *et al* where BMDC were stimulated with poly(I:C) for 0.5, 1, 2, 4, 6, 8, 12, 16 and 24 hours followed by RNA hybridization to an Affymetrix Mouse 430A 2.0 array. The applicable time points of 1, 4, 6, 12 and 24 hours from Amit *et al* were used in this comparison, where the 4 hour time point is compared to the 3 hour time point in this study as it displayed the most similar average gene expression values versus the 2 hour time point (data not shown). For each time point, all data reported by Amit *et al* (18858 probe sets) (x-axis) was plotted against the corresponding data from this study (y-axis). The scatter plots indicate that, except for 1 hour, all time points are positively correlated in both replicates (replicate data from this study was randomly paired with that of Amit *et al* for the scatter plots). Supplementary Table 3 below shows the array correlation coefficients between all replicates for all 18858 probe sets, and Supplementary Table 4 shows the improved correlations when using the sub-set of genes present in the 2-fold filtered data set (1,641 out of 2,142 were present in the Amit *et al* data).

Supplementary Table 3: Array correlation coefficients between each time point for Amit *et al* replicates (R) and experiments 1 and 2 from this study (E) for all 18,858 probe sets reported by Amit *et al*.

	R1 vs E1	*R1 vs E2*	*R2 vs E1*	*R2 vs E2*
1 hr	0.08	0.04	0.08	0.01
3-4 hrs	0.38	0.31	0.38	0.30
6 hrs	0.53	0.44	0.53	0.44
12 hrs	0.58	0.50	0.58	0.52
24 hrs	0.48	0.41	0.49	0.41

Supplementary Table 4: Array correlation coefficients between each time point for Amit *et al* replicates (R) and experiments 1 and 2 from this study (E) for the 1,641 genes found in the 2-fold filtered data set.

	R1 vs E1	*R1 vs E2*	*R2 vs E1*	*R2 vs E2*
1 hr	0.27	0.27	0.28	0.21
3-4 hrs	0.78	0.74	0.76	0.72
6 hrs	0.86	0.82	0.86	0.82
12 hrs	0.86	0.83	0.87	0.84
24 hrs	0.83	0.81	0.83	0.81
