# Supplementary material for: Dynamics of dendritic cell maturation are identified through a novel filtering strategy applied to biological time-course microarray replicates
Source: BMC Immunol. 2010 Aug 3;11:41. doi: 10.1186/1471-2172-11-41 (PMC2928180; doi:10.1186/1471-2172-11-41)
Supplement: Additional file 8 — Justification for removing the 1 hour time point. Discussion of a parallel analysis done using the 1 hour time point for filtering and consensus clustering, and the impact it had on the results. [file 1471-2172-11-41-S8.PDF]

Additional file 8: Justification for removing the 1 hour time point.

Pearson's correlation coefficient (PCC) had been calculated between each pair of replicate arrays before the execution of steps 2b and c, and it was determined that the 1 hour time point showed less consistency across experiments than the others (see Methods). This was done because both ED and PCC calculations between replicate profiles in steps 2b and c are sensitive to individual time point variation, so if one time point exhibits a large variation compared to other time points, the ED and PCC results would be affected.

To assess the effect the inconsistent 1 hour time point had on the rest of the consistency filtering and cluster analysis, these steps were performed with and without this time point, and the results were compared. Including the 1 hour time point in ED and PCC calculations of steps 2b and c increased the number of significantly correlated genes by 2% and 6% for the 2-fold and 4-fold filtered data, respectively. When the average standard deviation between replicate experiments is calculated for each time point, results showed that including the 1 hour time point increased the standard deviation for the other time points in the final filtered data (data not shown). This indicates that including the inconsistent 1 hour time point in the filtering process results in a gene list whose expression profiles exhibit additional variation. Likewise, cluster analysis (Figure 1, Step 3) was performed with and without the 1 hour time point to assess the effect on the resulting consensus clusters. Including the 1 hour time point in the cluster analysis process resulted in an increase of singleton clusters by 59% and 36% for the selected 4-fold and 2-fold filtered genes, respectively (a singleton cluster is a gene that didn't cluster with any other genes because its profile is an outlier). Additionally, a larger number of consensus clusters that were, on average, smaller in size were generated for both gene lists when the 1 hour time point was included (data not shown). Since inclusion of the inconsistent 1 hour time point introduced additional variation in the filtered gene lists and had a noticeably negative impact on the consensus clusters, it was not used in the remainder of the filtering and analysis process.
